# NCAM1 and GDF15 are biomarkers of Charcot-Marie-Tooth disease in patients and mice

**DOI:** 10.1093/brain/awac055

**Published:** 2022-02-11

**Authors:** Matthew J Jennings, Alexia Kagiava, Leen Vendredy, Emily L Spaulding, Marina Stavrou, Denisa Hathazi, Anika Grüneboom, Vicky De Winter, Burkhard Gess, Ulrike Schara, Oksana Pogoryelova, Hanns Lochmüller, Christoph H Borchers, Andreas Roos, Robert W Burgess, Vincent Timmerman, Kleopas A Kleopa, Rita Horvath

**Affiliations:** Department of Clinical Neurosciences, University of Cambridge, Cambridge, UK; Department of Neuroscience and Neuromuscular Disorders Centre, The Cyprus Institute of Neurology and Genetics, Nicosia, Cyprus; Peripheral Neuropathy Research Group, Department of Biomedical Sciences, Institute Born Bunge, University of Antwerp, Antwerp, Belgium; The Jackson Laboratory, Bar Harbor, ME, USA; Graduate School of Biomedical Sciences and Engineering, University of Maine, Orono, ME 04469, USA; Department of Neuroscience and Neuromuscular Disorders Centre, The Cyprus Institute of Neurology and Genetics, Nicosia, Cyprus; Department of Clinical Neurosciences, University of Cambridge, Cambridge, UK; Leibniz-Institut für Analytische Wissenschaften—ISAS—e.V, Dortmund, Germany; Peripheral Neuropathy Research Group, Department of Biomedical Sciences, Institute Born Bunge, University of Antwerp, Antwerp, Belgium; Department of Neurology, University Hospital Aachen, Aachen, Germany; Centre for Neuromuscular Disorders in Children, University of Duisburg-Essen, Essen, Germany; Directorate of Neurosciences, Royal Victoria Infirmary, Newcastle upon Tyne Hospitals, NHS Foundation Trust, Newcastle upon Tyne, UK; Division of Neurology, Department of Medicine, The Ottawa Hospital, Brain and Mind Research Institute and Children’s Hospital of Eastern Ontario Research Institute, University of Ottawa, Ottawa, Canada; Department of Neuropediatrics and Muscle Disorders, Medical Center–University of Freiburg, Faculty of Medicine, Freiburg, Germany; CNAG-CRG, Centre for Genomic Regulation, Barcelona Institute of Science and Technology, Barcelona, Spain; Segal Cancer Proteomics Centre, Lady Davis Institute, Jewish General Hospital, McGill University, Montreal, Quebec, Canada; Gerald Bronfman Department of Oncology, Jewish General Hospital, McGill University, Montreal, Quebec, Canada; Center for Computational and Data-Intensive Science and Engineering, Skolkovo Institute of Science and Technology, Moscow, Russia; Division of Neurology, Department of Medicine, The Ottawa Hospital, Brain and Mind Research Institute and Children’s Hospital of Eastern Ontario Research Institute, University of Ottawa, Ottawa, Canada; Department of Neurology, Heimer Institute for Muscle Research, University Hospital Bergmannsheil, Ruhr University Bochum, Bochum, Germany; The Jackson Laboratory, Bar Harbor, ME, USA; Graduate School of Biomedical Sciences and Engineering, University of Maine, Orono, ME 04469, USA; Peripheral Neuropathy Research Group, Department of Biomedical Sciences, Institute Born Bunge, University of Antwerp, Antwerp, Belgium; Department of Neuroscience and Neuromuscular Disorders Centre, The Cyprus Institute of Neurology and Genetics, Nicosia, Cyprus; Department of Clinical Neurosciences, University of Cambridge, Cambridge, UK

**Keywords:** Charcot-Marie-Tooth disease (CMT), mouse models, biomarker, serum, translational‌

## Abstract

Molecular markers scalable for clinical use are critical for the development of effective treatments and the design of clinical trials. Here, we identify proteins in sera of patients and mouse models with Charcot-Marie-Tooth disease (CMT) with characteristics that make them suitable as biomarkers in clinical practice and therapeutic trials.

We collected serum from mouse models of CMT1A (C61 het), CMT2D (*Gars*^C201R^, *Gars*^P278KY^), CMT1X (*Gjb1*-null), CMT2L (*Hspb8*^K141N^) and from CMT patients with genotypes including CMT1A (*PMP22*d), CMT2D (*GARS*), CMT2N (*AARS*) and other rare genetic forms of CMT. The severity of neuropathy in the patients was assessed by the CMT Neuropathy Examination Score (CMTES). We performed multitargeted proteomics on both sample sets to identify proteins elevated across multiple mouse models and CMT patients. Selected proteins and additional potential biomarkers, such as growth differentiation factor 15 (GDF15) and cell free mitochondrial DNA, were validated by ELISA and quantitative PCR, respectively.

We propose that neural cell adhesion molecule 1 (NCAM1) is a candidate biomarker for CMT, as it was elevated in *Gjb1*-null, *Hspb8*^K141N^, *Gars*^C201R^ and *Gars*^P278KY^ mice as well as in patients with both demyelinating (CMT1A) and axonal (CMT2D, CMT2N) forms of CMT. We show that NCAM1 may reflect disease severity, demonstrated by a progressive increase in mouse models with time and a significant positive correlation with CMTES neuropathy severity in patients. The increase in NCAM1 may reflect muscle regeneration triggered by denervation, which could potentially track disease progression or the effect of treatments.

We found that member proteins of the complement system were elevated in *Gjb1*-null and *Hspb8*^K141N^ mouse models as well as in patients with both demyelinating and axonal CMT, indicating possible complement activation at the impaired nerve terminals. However, complement proteins did not correlate with the severity of neuropathy measured on the CMTES scale. Although the complement system does not seem to be a prognostic biomarker, we do show complement elevation to be a common disease feature of CMT, which may be of interest as a therapeutic target.

We also identify serum GDF15 as a highly sensitive diagnostic biomarker, which was elevated in all CMT genotypes as well as in *Hspb8*^K141N^, *Gjb1*-null, *Gars*^C201R^ and *Gars*^P278KY^ mouse models. Although we cannot fully explain its origin, it may reflect increased stress response or metabolic disturbances in CMT. Further large and longitudinal patient studies should be performed to establish the value of these proteins as diagnostic and prognostic molecular biomarkers for CMT.

## Teaser

Using serum proteomics, Jennings *et al.* identify NCAM1 and GDF15 as sensitive and specific biomarkers for axonal and demyelinating forms of CMT in patients and four different mouse models. These markers could be used to support diagnosis and to monitor progression in clinical practice and therapeutic trials.

## Introduction

Hereditary motor and sensory neuropathies (HMSN) are a group of closely related disorders affecting the motor and sensory neurones of the peripheral nervous system. With an estimated prevalence of 1 in 2500,^1,2^ Charcot-Marie-Tooth disease (CMT) is among the most common hereditary neuromuscular disorders. CMT is categorized into demyelinating and axonal subtypes, based on the primary mechanism of degeneration. In demyelinating CMT (CMT1), degeneration begins with dysfunction of the Schwann cells forming the myelin sheath, leading to decreased nerve conduction velocities and secondary axonal degeneration, whilst in axonal CMT (CMT2) the degeneration of the axon occurs primarily. The most common genetic form of CMT is CMT1A, accounting for around half of all CMT patients and 70% of CMT1 patients in Western Europe.^3^ CMT1A is most commonly caused by a 1.5 Mb tandem duplication on chromosome 17p11.2-p12 that includes the peripheral myelin protein 22 gene (*PMP22*, here referred as *PMP22*d),^4,5^ a protein critical to the synthesis of myelin layers ensheathing the peripheral axons. Variants in other genes involved in myelin proteins (e.g. *MPZ*) or other factors required for the maintenance of the myelin sheet can also lead to demyelinating CMT.^6^ The second most common form of CMT is CMT1X, caused by mutations in the X chromosomal *GJB1* gene.^7^ With an X-linked dominant inheritance, CMT1X affects both sexes, but typically takes a more progressive, severe and predominantly demyelinating form in males; while it is stable, milder and often axonal in females.^8^ CMT2 is caused by a diverse range of genes with physiological functions commonly affecting mitochondrial dynamics (*MFN2*, *DNM2*, *GDAP1*), molecular chaperones (*HSPB1*, *HSPB8*) and mutations in the aminoacyl-tRNA synthetase (aaRS) genes (*GARS*, *AARS*, *YARS*, *KARS*, *MARS*, *WARS*).^9^

Several therapies have been trialled in the past for CMT, including major human trials of ascorbic acid^10^ and PTX3003 (combination therapy of baclofen, naltrexone and sorbitol).^11^ Ascorbic acid has a robust body of evidence showing a lack of efficacy, whilst PTX3003 is under investigation in a phase III clinical trial (NCT04762758). Additionally, there are many smaller studies of experimental treatments in various forms of CMT, which when taken together indicate that some therapies can be useful in specific genotypes based on the molecular mechanism.^12,13^ Some genetic subtypes of inherited peripheral neuropathies are caused by enzyme defects leading to accumulation of toxic metabolites. An increase of serum deoxysphingolipids has been observed in patients with *SPTLC1* mutations,^14^ while high sorbitol levels were detected in *SORD-*related CMT2.^15^ These make powerful biomarkers in these selected forms of CMT, which could be used to follow the molecular effect of targeted treatments with L-serine or aldose reductase inhibitors.^14,15^ However, most known forms of CMT are not caused by a specific metabolic disturbance and require general non-metabolic biomarkers in order to monitor severity and determine the efficacy of treatments such as repurposed drugs, small molecules or gene therapies.

Plasma neurofilament light chain (NEFL), a previously reported biomarker of several neurodegenerative diseases,^16^ has been shown to be increased in mouse models and patients with various genetic forms of CMT. Plasma NEFL correlates with disease severity^17^; however it has a low diagnostic selectivity (71% sensitivity and 75% specificity).

Additional promising biomarkers have been suggested for CMT1A. *PMP22* transcripts were strongly increased in rodent models and patients, without correlation with neuropathy progression.^18,19^ Multiplexed immune-assay identified elevated plasma TMPRSS5 in CMT1A patients (*n* = 47), without correlation with disease severity.^20^ It has recently been proposed that a set of micro RNAs (*miR-206*, *miR-133a* and *miR-223-3p*) are also candidate biomarkers for CMT1A, as they may reflect Schwann cell processes that underlie the pathogenesis of the disease. Furthermore, a recent metabolomics approach identified that CMT1A is associated with a metabolic state resembling inflammation and sarcopenia, suggesting that it might represent a potential target to prevent the nerve and muscle wasting phenotype in these patients.^21^

An age-adjusted combination of cutaneous *GSTT2* and *CTSA* mRNA levels differentiated CMT1A patients from controls,^18^ which was replicated in a subsequent larger (*n* = 266) international multicentre study.^22^ Furthermore, *GSTT2* and *CTSA* mRNA, used in combination with *PPARG*, *CDA*, *ENPP1* and *NRG1* transcripts, may be predictive of CMT1A disease progression.^22^ Many potential biomarkers are relevant only for the CMT1A subtype, and other markers have relatively low diagnostic sensitivity and unclear prognostic or progression-monitoring capacity, therefore a need remains to identify further accessible biomarkers of CMT.

Profilin 2 (PFN2) and guanidinoacetate *N*-methyltransferase (GAMT) were downregulated in lymphoblastoid cells of patients with axonal CMT.^23^ Reduced PFN2 expression was also detected in induced pluripotent stem cell (iPSC)-derived motor neurons and sciatic nerves of CMT2 mice; however, GAMT levels were unchanged in motor neurons and CMT2 mouse-derived sciatic nerves.^23^

With many new therapies nearing clinical trials, the lack of molecular biomarkers remains a key limitation in assessing the efficacy of treatments in this slowly progressive disease.^12^ To identify novel serum biomarkers common across CMT subtypes, we performed targeted mass spectrometry using serum of a cohort of 55 patients and four mouse models with different genetic forms of CMT. Combining the results in patients and mice led to the identification of NCAM1 and GDF15 as powerful biomarkers of CMT, and we found widespread dysregulation of the complement inflammatory system.

## Materials and methods

### Patients

We studied a cohort of 55 patients from the inherited peripheral neuropathy clinic at the Newcastle upon Tyne Hospitals NHS Foundation Trust.^24^ Healthy control serum was taken from unaffected family members of the patients and from age-matched healthy volunteers. Informed consent was obtained from each participant (Yorkshire and The Humber, Leeds Bradford, REC13/YH/0310). As a disease control, we also studied serum from 11 patients with GNE myopathy presenting with distal muscle wasting in a similar age to CMT (North East, Newcastle and North Tyneside 1, REC13/NE/0123) and patients with Becker or Duchenne muscular dystrophy (BMD/DMD) (08/H0906/28+5). GNE myopathy was used as a disease control of slowly progressive distal muscle weakness and wasting in young adults (similar pattern to CMT), while BMD/DMD represents progressive proximal weakness and wasting from a young age. The serum samples were stored in the Newcastle Biobank of the MRC Centre for Neuromuscular Diseases (NRES Committee North East, Newcastle and North Tyneside 1 (08/H0906/28+5).^25^ Charcot-Marie-Tooth Examination Scores (version 2, CMTES)^26^ were available for 44 of the 55 CMT patients enrolled. Patient serum was collected during routine appointments at the clinic from patients at various stages of disease progression.

### Mouse models and serum collection

Mouse models of four neuropathy genes were used in our analysis: *PMP22*, *GJB1*, *HSPB8* and *GARS*. Heterozygous Tg(*PMP22*)C61Clh model (C61 het) carries four copies of the human *PMP22* along with normal murine *Pmp22*, resulting in PMP22 overexpression, demyelination and neuropathy.^27^ Histologically, C61 het mice initially develop normal myelin and then show consecutive demyelination and axonal loss with onion bulb formation, while their motor nerve conduction velocities (MNCVs) are between 20–30 m/s.^27,28^ Thus, the C61 het mouse is an appropriate model for CMT1A, similar to the rat model.^18^

Homozygous *Gjb1*-null mice were obtained from the European Mouse Mutant Archive, originally generated by Klaus Willecke (University of Bonn) by in-frame insertion to exon 2 of *Gjb1*.^26,29^ Although many patients with CMT1X carry missense mutations in *GJB1*, most of these variants result in loss of the protein, while others lead to trafficking or channel defects, but all are loss-of-function in some way; therefore, the *Gjb1* null mice is representative for the *GJB1*-related human disease.^30^ Furthermore, there is robust treatment-responsive elevation of NEFL in this model.^31^

*Hspb8* ^K141N^ knock-in mice were developed by Vincent Timmerman (University of Antwerp).^32^ CMT2D (*GARS*) was modelled using 2 knock-in models carrying the C201R (ENU mutagenesis) and P278KY missense mutations in murine *Gars*, characterized by Elizabeth Fisher and Robert Burgess.^33,34^ All mice are bred on a C57BL/6 background, and wild-type control littermates were available for combinations of age and mutational status. *Gars*^P278KY^ and *Gars*^C201R^, and wild-type littermate blood was collected by submanibular puncture into serum collection tubes, allowed to clot for 15–30 min and centrifuged at 5000*g* for 10 min at room temperature. *Hspb8*^K141N^ mouse blood was collected from the heart at death; Gjb1-null and C61 het mouse blood was collected from the retro-orbita;^35^ and serum was separated using MiniCollect® CAT serum separator tubes by centrifugation at 3000*g* for 10 min at room temperature. All serum was stored at −80°C until use.

### Comparative proteomic quantification studies in serum

Mouse and human serum samples were analysed by the University of Victoria-Genome BC Proteomics Centre by LC/MRM-MS (liquid chromatography-multiple reaction monitoring mass spectrometry) using SIS (stable isotope-labelled standard) peptides. Mouse and human peptide panels were of 270 and 375 peptides (Supplementary Tables 1 and 2), respectively, selected as peptides previously validated in LC-MRM experiments according to the Office of Cancer Clinical Proteomics Research guidelines of assay development.^36^ For details of mass spectrometry and analysis, see the Supplementary material.

### ELISA studies

Identified lead markers from proteomics were confirmed by ELISA. We also used ELISA to study the established mitochondrial biomarker GDF15. ELISA quantification was performed for mouse Ncam1 (Rockland, KOA0409) and Gdf15 (R&D Systems, MGD150), human NCAM1 (R&D, DY2408) and GDF15 (R&D Systems, DGD150) according to manufacturer’s instructions.

### Immuno-labelling of human muscle sections and microscopy

Ten micrometre-thick muscle sections were stained with antibodies targeting C1q, C3 or NCAM1 alongside antibodies targeting spectrin/α-II-spectrin and α-bungarotoxin-AlexaFluor647 and imaged by confocal microscope. For full details of labelling and confocal imagine, see the Supplementary material.

### Cell-free mitochondrial DNA quantification

Serum circulating cell-free mitochondrial DNA (ccf-mtDNA) was quantified by first isolating DNA from 100 µl of serum using Qiagen DNeasy blood DNA extraction kit (69504), according to manufacturer’s instructions. Duplexed Taqman (iTaq, Bio-Rad) amplification of the mitochondrially-encoded genes *MT-ND1* and *MT-ND4* is then undertaken using targeted probes as previously described.^37,38^

### Statistical analysis

Mouse serum samples were assessed for statistical signficance by *t*-test, and thresholds set at *P* < 0.05 for two time points or individual variant (for *Gars*^mut^) in two different models. For patient serum samples, statistical analysis of protein peptide abundances was performed by *t*-test of CMT versus control, CMT1 versus control, CMT2 versus control and CMT versus GNE myopathy or BMD/DMD. All peptides for which *P* < 0.05 of any patient group after Hochberg correction were selected for further statistical visual analysis and further consideration. Linear regression analysis was used to identify correlation between protein abundance and neuropathy severity as assessed by the CMTES score.^26^ All statistical analyses were performed using custom R scripts, using base R statistical test functions.

### Data availability

The authors confirm that the data supporting the findings of this study are available within the article and its Supplementary material.

## Results

### Study design

Serum protein peptides were analysed by targeted MRM-MS quantification in serum samples of the four mouse models and the CMT patient cohort (Supplementary Tables 1 and 2).

**Figure 1:**
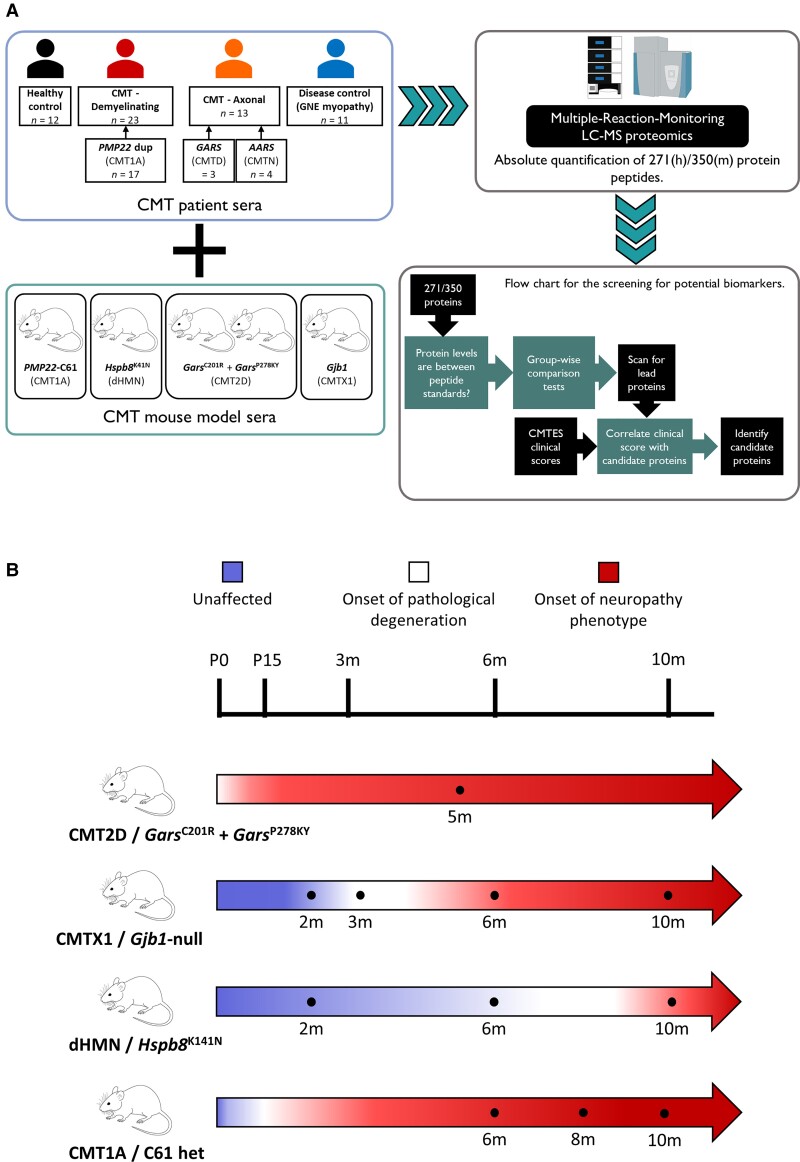
**Study design.** (**A**) Schematic of mass spectrometry proteomic serum protein biomarker identification in human and mouse, and statistical analysis framework. (**B**) Progression of pathological and symptomatic neuropathy of mouse models, and time points of serum collection.

Sera was collected from *Gars*^C201R^ and *Gars*^P278KY^, *Gjb1*-null, *Hspb8*^K141N^ and C61 het (CMT1A, *PMP22*d model) mice alongside wild-type littermate controls. Serial serum samples were taken at different time points, corresponding to different phases of neuropathy (Fig. 1B). Fifty-one proteins were significantly altered in at least two time points of each gene (or both gene variants for *Gars*^mut^) in one or more of the four mouse models, but only seven were shared across more than one model. This identified complements C1q-B and C1q-C, neural cell adhesion molecule 1 (Ncam1), proteasome subunit beta type 4 (Psmb4), pyruvate kinase R/L (Pklr), pyruvate kinase M1/M2 (Pkm) and thyroxine-binding protein (Serpina7) as commonly elevated proteins in CMT mouse models (Supplementary Table 1).

Serum was also collected from 41 genetically confirmed CMT patients, and 12 healthy age-matched controls (Fig. 1A). The CMT1 patient group was of mixed genotypes including *PMP22*d (CMT1A; *n* = 21), *GJB1* (CMT1X, *n* = 1) and *MPZ* (CMT1B, *n* = 1). CMT2 patients carried pathogenic variants in *GARS* (CMT2D; *n* = 4), *AARS* (CMT2N; *n* = 6), *DHTKD1* (CMT2Q, *n* = 3), *DNM2* (CMT2M, *n* = 1), *DYNC1H1* (CMT2O, *n* = 1), *MFN2* (CMT2A, *n* = 1), *MME* (CMT2T, *n* = 1), *RAB7* (CMT2B, *n* = 1) and in one patient the genetic cause of CMT2 remained unknown (*n* = 1). Patients were grouped into CMT1 (*n* = 23) and CMT2 (*n* = 18) groups as well as into specific subtypes for some selected genotypes such as *PMP22*d (*n* = 17) and a combined *GARS* and *AARS* (*n* = 10) genotype due to common clinical presentation and likely shared pathophysiology.^39,40^

Group-wise comparisons were subjected to individual Hochberg correction for multiple comparisons. Six proteins were significantly elevated in CMT1 patient sera: multiple complement pathway proteins C1q-A, C1q-B, C1r, C3 and factor H (CFH), along with the complement inhibitor vitronectin (VTN). Four proteins were significantly elevated in CMT2 patient sera: apolipoprotein E (APOE), complements C3 and CFH and kininogen 1 (KNG1). Only complements C3 and CFH were significantly elevated in both axonal and demyelinating CMT, though without Hochberg-correction this would also include APOE, KNG1, inter alpha trypsin inhibitor heavy chain H2 (ITIH2), and VTN.

Therefore, patients replicate elevation of C1q-B identified in mouse models, and demonstrated elevation of other complement pathway proteins. SERPINA7 was detected in patient serum, but was similar between controls and patient groups (Supplementary Table 2). NCAM1, PSMB4, PKLR, PKM did not have orthologous human peptides available and therefore could not be determined in patient sera. NCAM1 has previously been implicated in neurodegenerative diseases,^41–43^ and therefore was prioritized for quantification by ELISA in patient sera. Of the other proteins identified in the patient screen: APOE, KNG1, ITIH2 and VTN were not elevated in two time points for any mouse model.

### Serum NCAM1 correlates with neuropathy progression

**Figure 2:**
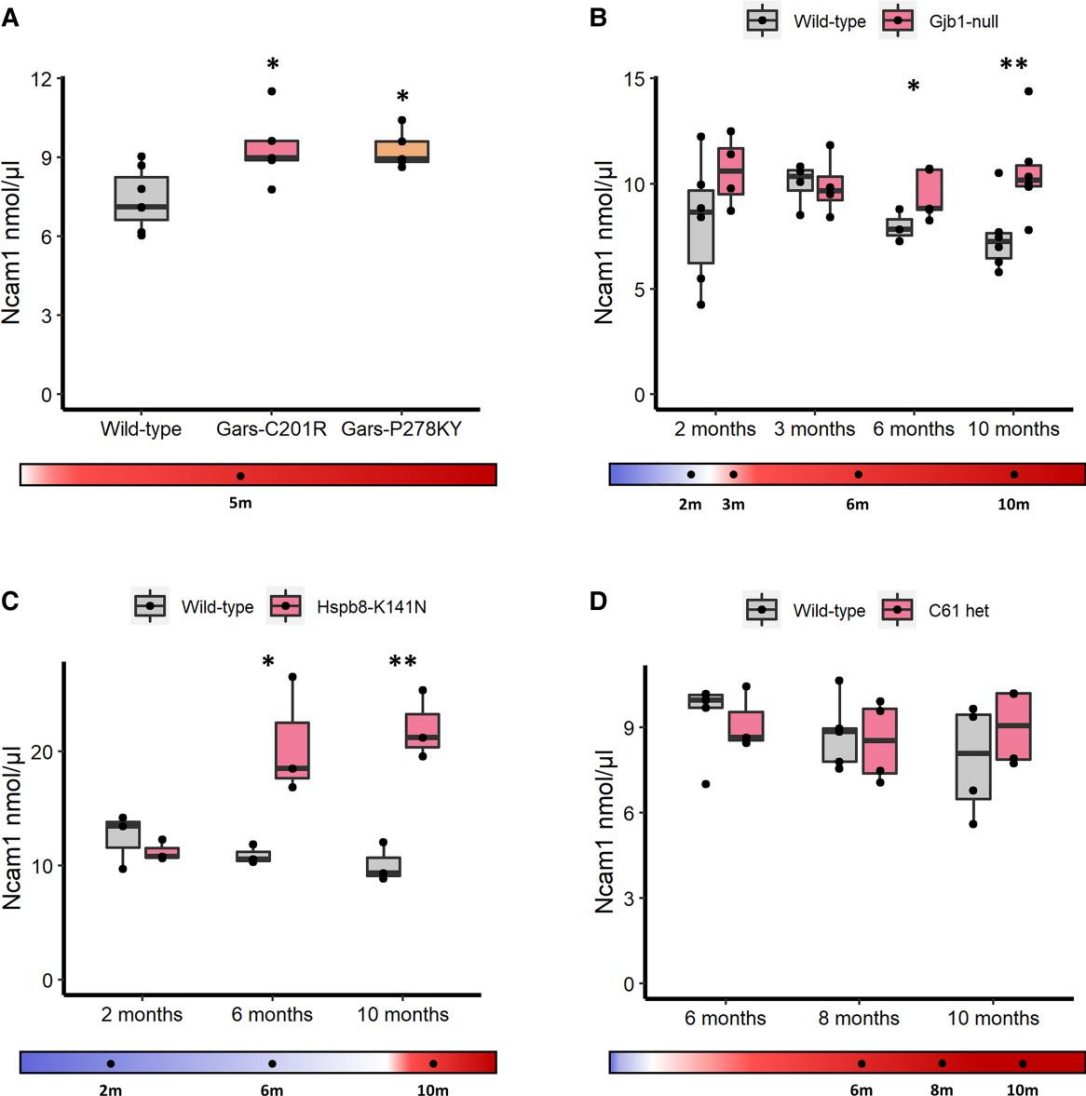
**Serum Ncam1 analysis.** (**A**) control (*n* = 7), *Gars*^C201R^ (*n* = 5) and *Gars*^P278KY^ (*n* = 5);(**B**) control (*n* = 6/4/3/6) versus *Gjb1*-null (*n* = 4/4/5/6); (**C**) control (*n* = 3/3/3) *Hspb8*^K141N^ (*n* = 3/3/3); (**D**) control (*n* = 5/5/4) and C61 het (*n* = 3/4/4) mouse models, determined by ELISA quantification. Statistical significance indicated by Student’s *t*-test, where *P < 0.05, **P < 0.01.

Proteomic screening identified Ncam1 as elevated in *Gars*^C201R^, *Gars*^P278KY^, *Gjb1*-null and *Hspb8*^K141N^ mice compared to controls (Fig. 2). *Gars*^C201R^ and *Gars*^P278KY^ mice exhibit axon loss and display a behavioural neuropathy phenotype from around 1 month of age, and the elevation of Ncam1 was present at 5 months of age in both mutant models (Fig. 2A). In *Gjb1*-null mice, significant elevation of Ncam1 is evident from 6 months, after the onset of the neuropathy which occurs at 3 months of age (Fig. 2B). Ncam1 is elevated from 6 months of age in *Hspb8*^K141N^ mice, concomitant with the development of early axonal loss implied by decreased compound motor action potentials.^32^ No significant difference in serum Ncam1 was detected in the C61 het mice at 2 and 10 months of age, despite this mouse model presenting with functional impairment (reflective of axon loss) from 6 months of age. This may reflect the small sample size when combined with a variable ratio of demyelination versus axonal loss, associated with a variable degree of PMP22 expression in this model.

NCAM1 was also determined by ELISA to confirm the proteomics results (Supplementary Fig. 1). Increased serum NCAM1 was detected in *Hspb8*^K141N^ mice from 10 months, *Gjb1*-null mice from 6 months and in both *Gars*^C201R^ and *Gars*^P278KY^ models at 5 months of age (Supplementary Fig. 1). The delay of a statistically significantly difference to 10 months when measured by ELISA is likely to be a result of lower assay sensitivity, which may also explain the less pronounced degree of increase in the 10-month mice. Similar to proteomic data, no significant increase was identified in C61 het mouse sera (Supplementary Fig. 1). Together, these data indicate that NCAM1 is increased across different CMT mouse models compared to littermate controls, with the difference becoming more noticeable with increasing age and neuropathy severity. Across control animals there was a progressive decrease in NCAM1 levels with age, whilst in mutant animals NCAM levels generally increased with age.

**Figure 3:**
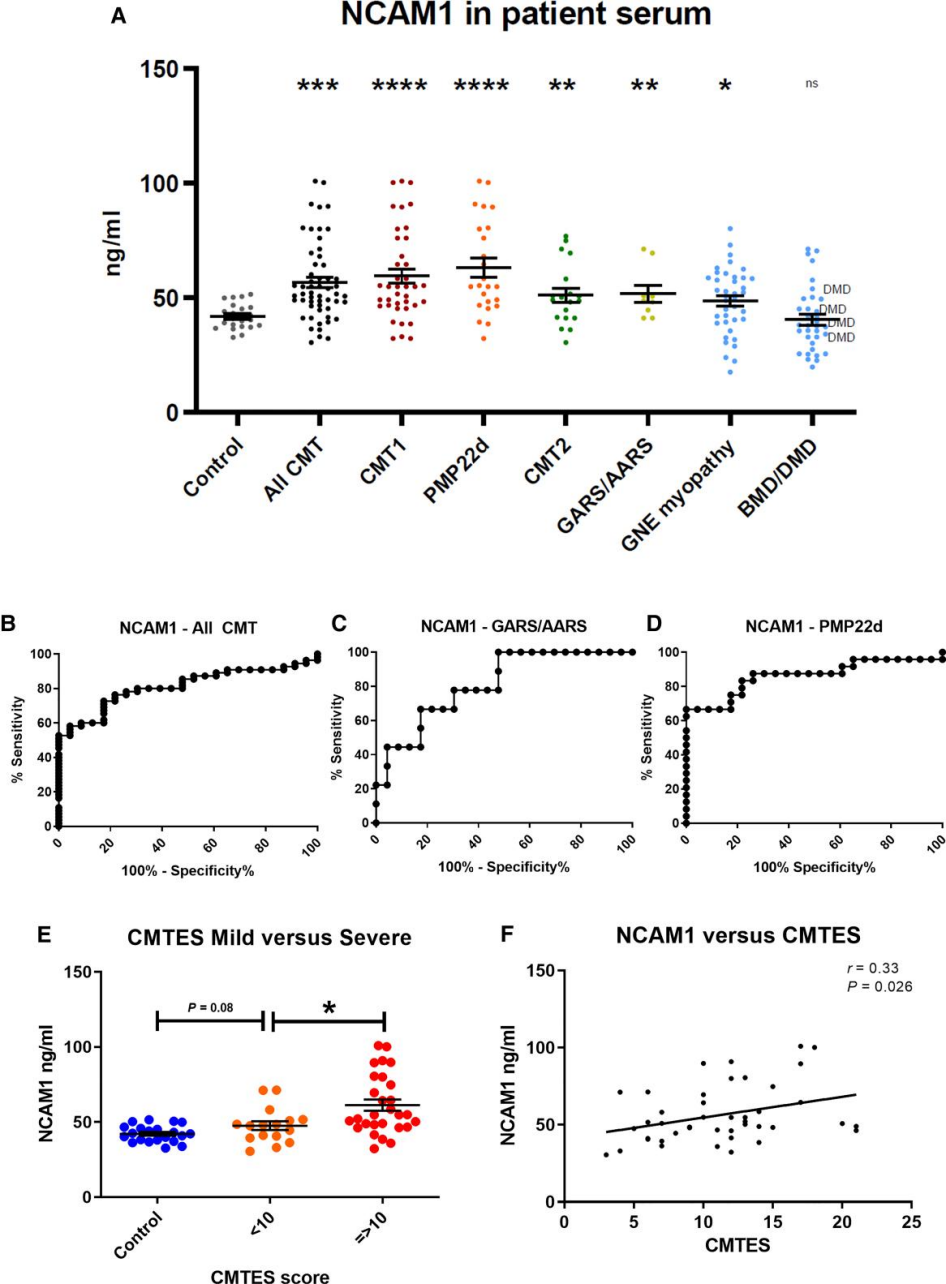
**NCAM1 expression in patient sera, determined by ELISA quantification.** (**A**) NCAM1 expression in healthy controls (*n* = 23); all CMT (*n* = 55), CMT1 (*n* = 39), *PMP22*d (CMT1A) (*n* = 24), CMT2 (*n* = 19), *GARS*/*AARS* (*n* = 9) neuropathy patients; and GNE myopathy patients (*n* = 39). NCAM1 ROC curves for: (**B**) CMT (all) versus control, AUC = 0.748 (95% CI; 0.627–0.869); (**C**) *GARS*/*AARS* versus control, AUC = 0.747 (95% CI; 0.578–0.914); (**D**) PMP22d versus control, AUC 0.797 (95% CI; 0.661–0.932). (**E**) NCAM1 in control (*n* = 23), mild (CMTES < 10, *n* = 16) and severe patients (CMTES ≥ 10, *n* = 28). (**F**) NCAM1 versus CMTES in CMT patients (*n* = 44). Statistical significance indicated by Student’s *t*-test, where *P < 0.05, **P < 0.01, ***P < 0.001, ****P < 0.0001.

NCAM1 was quantified in human serum samples by ELISA, since human NCAM1 peptides were not available for the MRM analysis. ELISA quantification showed a 22.2% increase in expression of NCAM1 in CMT patients compared to controls (*P* = 0.0004), with 36.1% increase in CMT1A patients (*P* = 0.0028) and 11.6% increase in patients with mutations in *GARS/AARS* (*P* = 0.0318) (Fig. 3A). With threshold set at 44.34 ng/ml NCAM1 distinguishes CMT patients from controls in this study with a sensitivity of 78% and a specificity of 70%. To assess the diagnostic capacity across different NCAM1 thresholds, receiver operated characteristic (ROC) curves were generated to quantify the diagnostic capacity of given marker as the classification threshold is varied (Fig. 3B–D). The ROC area-under the curve (AUC) for NCAM1 to identify CMT patients from age- and sex-matched healthy controls was 0.748 (95% CI; 0.6268–0.869), for *GARS*/*AARS*-related CMT versus control AUC was 0.7467 (95% CI; 0.578–0.914) and for CMT1A 0.797 (95% CI; 0.661–0.9324), respectively (Fig. 3B–D). Serum NCAM1 concentrations are significantly (*P* = 0.016) higher in CMT patients with severe neuropathy (CMTES ≥ 10), at 61.3 ng/ml, compared to those with mild neuropathy (CMTES < 10), at 47.6 ng/ml; an increase of 28.7% (Fig. 3E). Furthermore, there is a significant direct correlation between serum NCAM1 levels and CMTES score itself (*r* = 0.33, *P* = 0.026) even within this relatively small group (Fig. 3F). NCAM1 in BMD/DMD patients was not significantly different to controls, while in GNE myopathy there was a small increase compared to controls, but still significantly lower than in CMT patients (*P* = 0.032). NCAM1 did not correlate with age in either patients or controls (Supplementary Fig. 2).

### The inflammatory complement system is activated in CMT patients and mouse models

**Figure 4:**
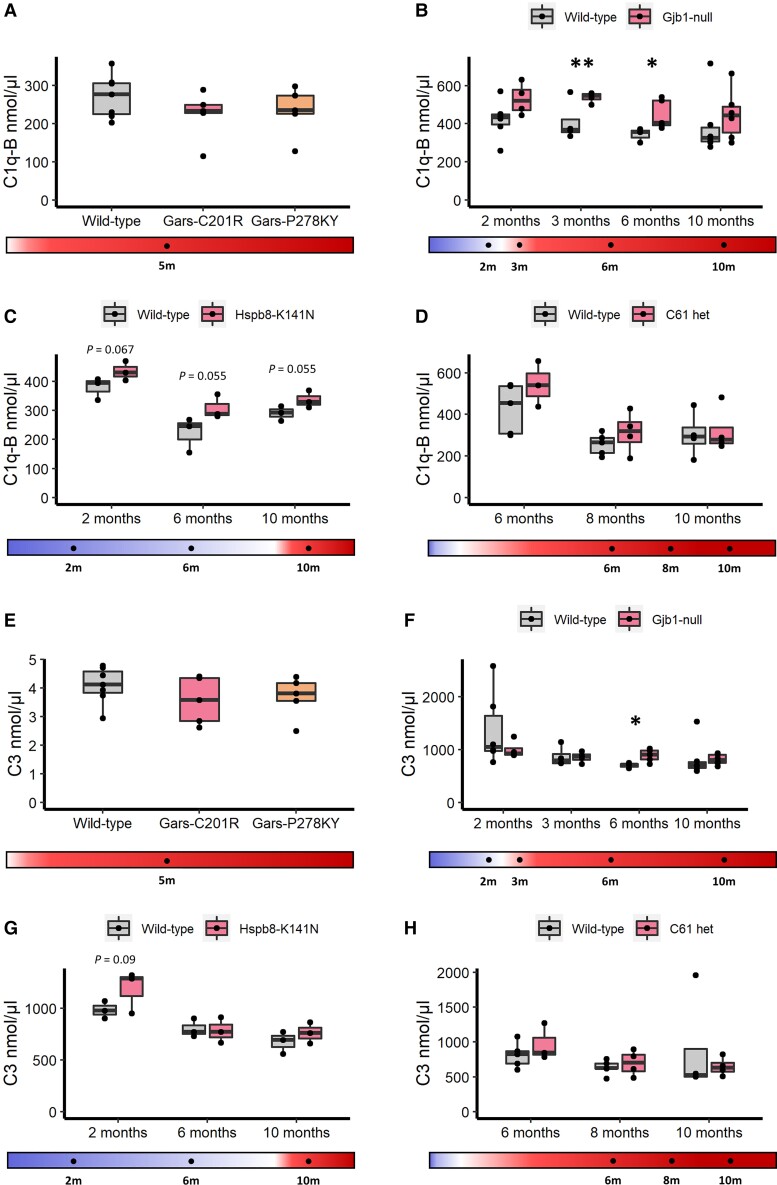
**Serum complement C1q-B analysis in mice.** (**A**) control (*n* = 7) *Gars*^C201R^ (*n* = 5) and *Gars*^P278KY^ (*n* = 5); (**B**) control (*n* = 6/4/3/6) and *Gjb1*-null (*n* = 4/4/5/6); (**C**) control (*n* = 3/3/3) and *Hspb8*^K141N^ (*n* = 3/3/3); (**D**) control (*n* = 5/5/4) and C61 het (*n* = 3/4/4) mouse models. Serum complement C3 in: (**E**) control (*n* = 7) *Gars*^C201R^ (*n* = 5) and *Gars*^P278KY^ (*n* = 5); (**F**) control (*n* = 6/4/3/6) and *Gjb1*-null (*n* = 4/4/5/6); (**G**) control (*n* = 3/3/3) and *Hspb8*^K141N^ (*n* = 3/3/3); (**H**) control (*n* = 5/5/4) and C61 het (*n* = 3/4/4) mouse models, by MRM-mass spectrometry. Statistical significance indicated by Student’s *t*-test, where *P < 0.05, **P < 0.01.

Multiple members of the inflammatory complement system were elevated in *Gjb1*-null and *Hspb8*^K141N^ mice compared to controls. Most consistent were complement C1q-B and C3. C1q-B, along with C1q-A and C1q-C are protein subunits of C1q. In *Gjb1*-null mice, C1q-B was significantly elevated at 3 and 6 months, corresponding to the onset of myelin abnormalities at 3 months in this model while a non-significant elevation at 10 months was observed (Fig. 4B). In *Hsbp8*^K141N^ mice, there is a near-significant elevation (*P* < 0.1) at all three time points, from prior to any deficit in nerve conduction through to the age with clinically manifesting neuropathy symptoms (Fig. 4C). C1q-B was stable in *Gars*^C201R^, *Gars*^278KY^ or C61 het mouse models compared with controls (Fig. 4A and D).

Complement C3 was also increased in CMT mouse models, following a similar pattern. In *Gjb1*-null mice C3 was elevated at the post-symptomatic 6- and 10-month time points (Fig. 4E), but was statistically significant only at 6 months (Fig. 4F). In *Hspb8*^K141N^, increased C3 expression compared to control was detectable at 2 and 10 months of age (Fig. 4G), corresponding to both pre-symptomatic and symptomatic phases. In contrast, C3 was stable in *Gars*^C201R^, *Gars*^278KY^ (Fig. 4E) or C61 (Fig. 4H) het mouse models compared with controls.

**Figure 5:**
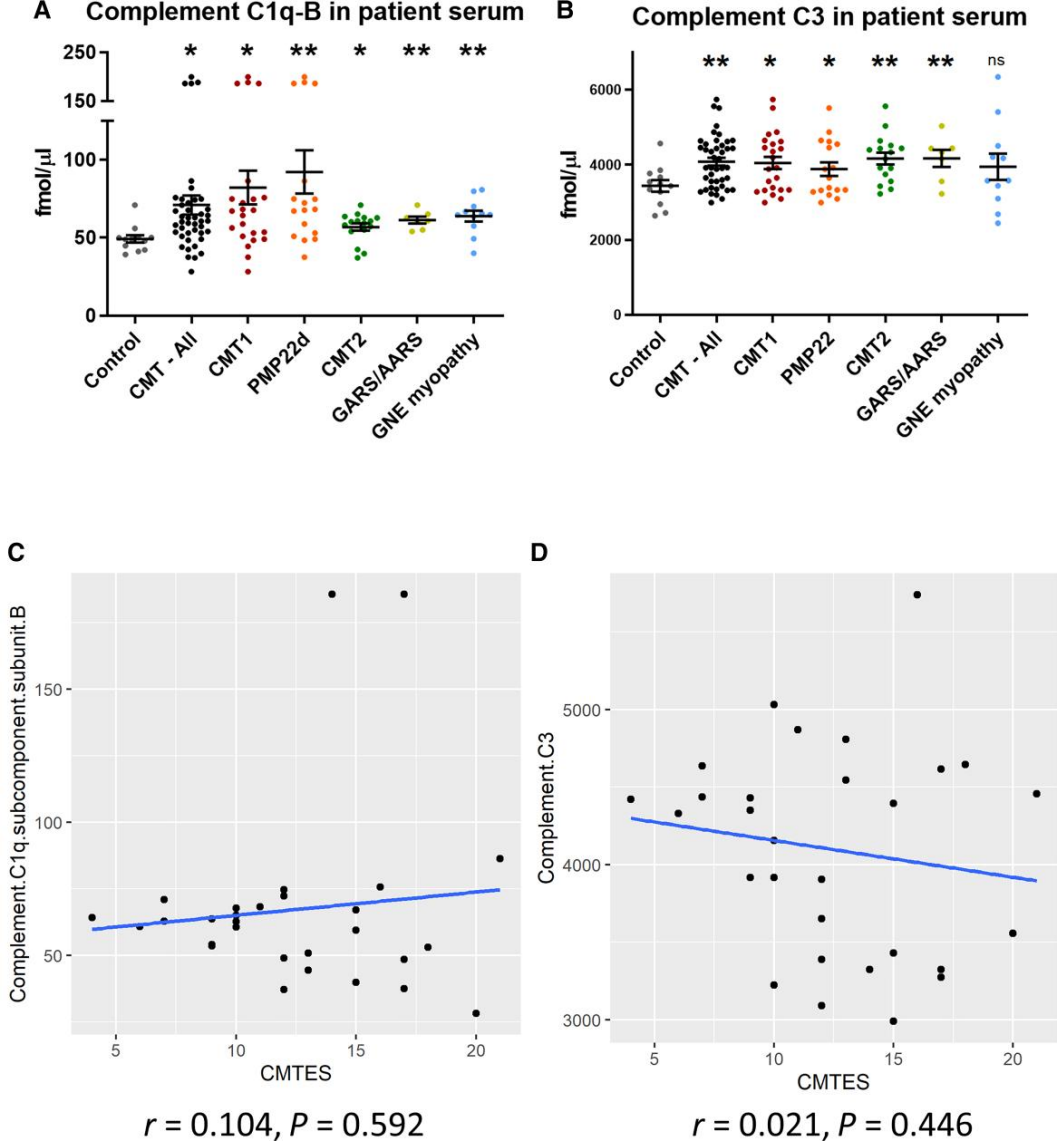
**Serum complement C1q-B analysis in patients.** Healthy controls (*n* = 12); all CMT (*n* = 43), CMT1 (*n* = 23), *PMP22*d (*n* = 17), CMT2 (*n* = 16) and *GARS*/*AARS* (*n* = 7) patients; and GNE myopathy patients serum complements (**A**) C1q-B and (**B**) C3. Correlation with CMTES of serum complements (**C**) C1q-B (*n* = 28) and (**D**) C3 (*n* = 28). Determined by MRM-mass spectrometry. Statistical significance indicated by Student’s *t*-test, where *P < 0.05, **P < 0.01.

In CMT patient sera, there was a significant increase in serum complement system proteins compared to controls. C1q and C3 were elevated in both axonal and demyelinating CMT patients sera, as well as GNE myopathy, compared with controls (Fig. 5A and B). Other complement pathway proteins such as C1r, C8-β, complement factor B, and complement factor I were all elevated in both axonal and demyelinating forms of CMT (Supplementary Table 2). Neither C1q, C3 or the other elevated complement proteins has a significant correlation with CMTES in CMT patients (Fig. 5C and D and Supplementary Fig. 4). C1q and C3 did not correlate with patient age (Supplementary Fig. 5).

### GDF15 is a highly sensitive diagnostic biomarker of CMT

**Figure 6:**
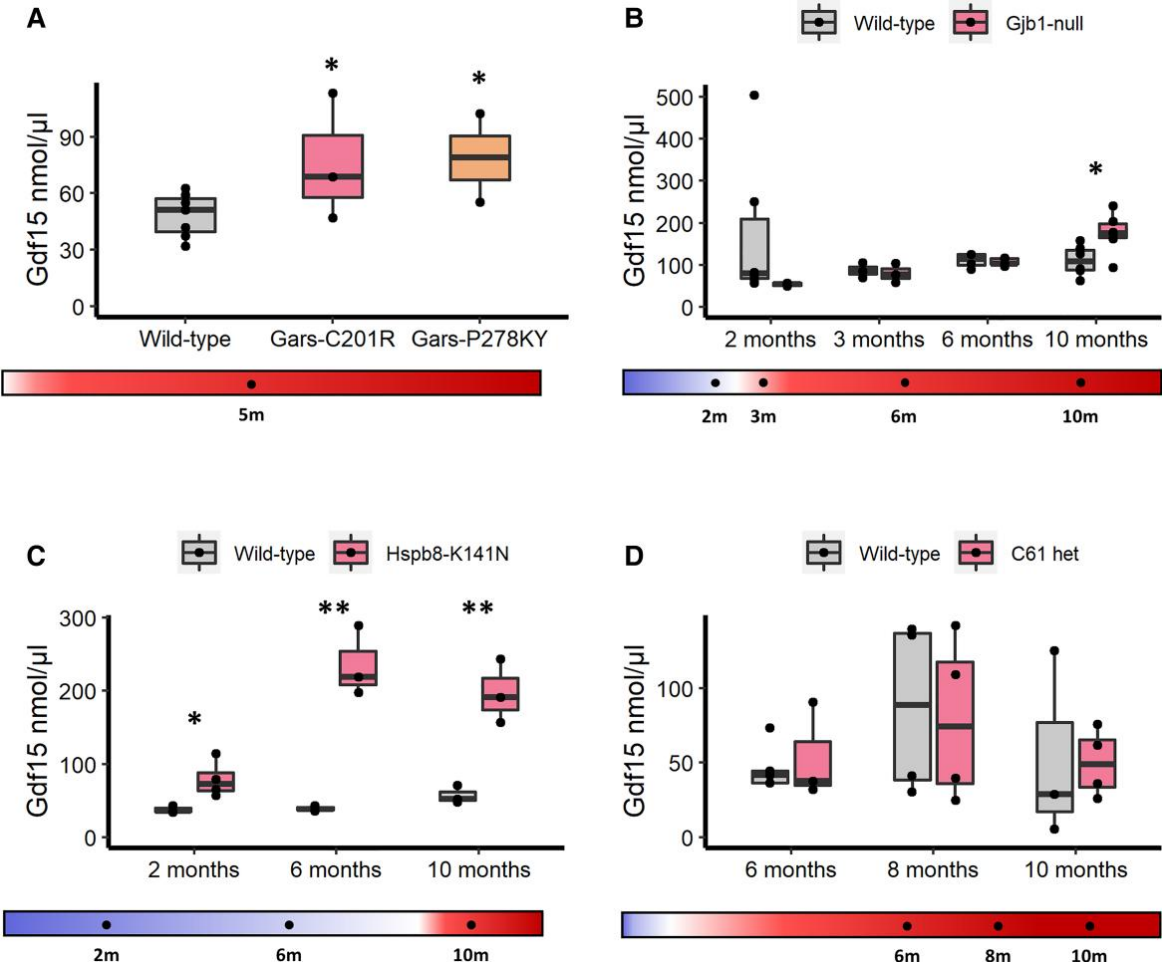
**Serum Gdf15 analysis in mice.** (**A**) Controls (*n* = 7), *Gars*^C201R^ (*n* = 3) and *Gars*^P278KY^ (*n* = 2); (**B**) controls (*n* = 6/3/4/6) and *Gjb1*-null (n = 4/3/5/6); (**C**) control (n = 3/3/3) and *Hspb8*^K141N^ (*n* = 4/3/3); (**D**) controls (*n* = 5/4/3) and C61 het (*n* = 3/4/4) mouse models determined by ELISA quantification. Statistical significance indicated by Student’s *t*-test, where *P < 0.05, **P < 0.01.

GDF15 was not a protein included in the targeted proteomic screening but is established already as a biomarker for mitochondrial disease, recently indicated to be elevated in *Gars* mice as well as in patients carrying pathogenic variants in *GARS*, *AARS* and *PMP22*d.^44^ Therefore, we investigated whether it may have biomarker characteristics in our CMT cohort. As expected, we found that *Gars*^C201R^ and *Gars*^P278KY^mice have elevated GDF15 at 5 months of age, in line with increased gene expression in these models (Fig. 6A). *Gjb1*-null mice had significantly elevated GDF15 only at 10 months, with no changes observed at younger time points (Fig. 6B). *Hspb8*^K141N^ mice had significantly elevated GDF15 from 2 months of age, the earliest time point, prior to symptoms onset, rising to even higher levels at 6 and 10 months of age (Fig. 6C). No change was determined for C61 het mice (Fig. 6D).

**Figure 7:**
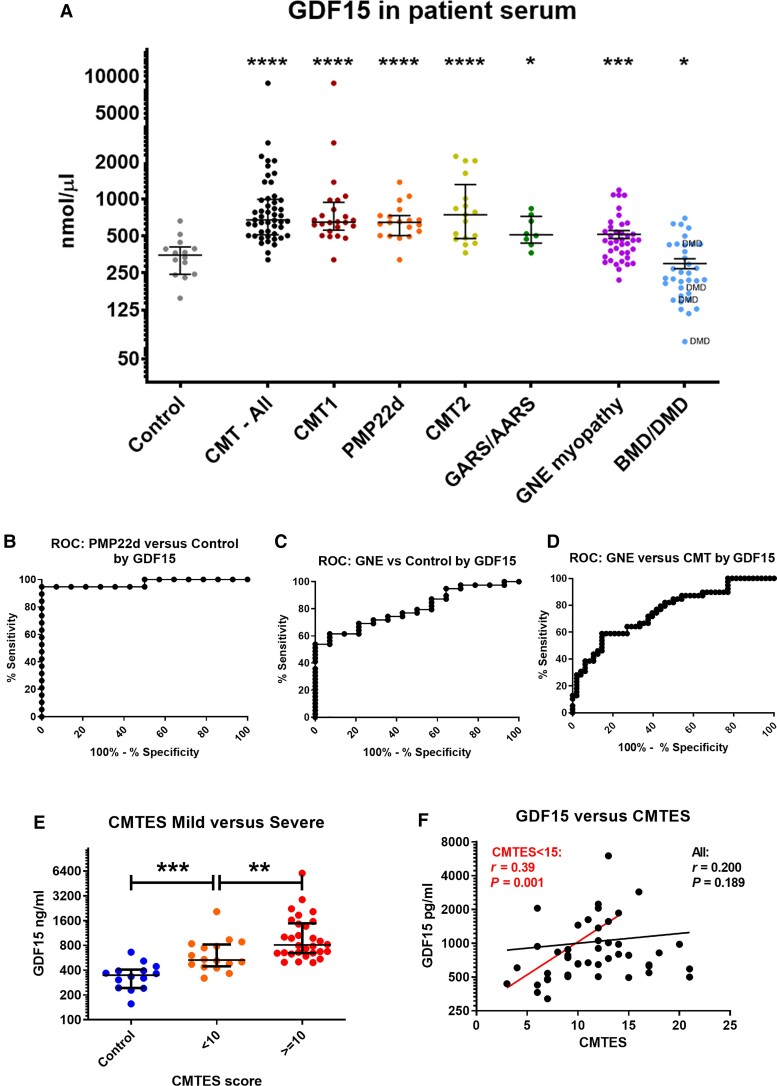
**GDF15 expression in patient sera determined by ELISA quantification.** (**A**) GDF15 expression in healthy controls (*n* = 14); all CMT (*n* = 49), CMT1 (*n* = 24), *PMP22*d (*n* = 19), CMT2 (*n* = 17) and *GARS*/*AARS* (*n* = 8) neuropathy patients; GNE myopathy (*n* = 39), BMD (*n* = 30) and DMD (*n* = 4) patients. GDF15 ROC curves for: (**B**) CMT (all) versus control, AUC = 0.972 (95% CI; 0.936–1); (**C**) *GARS*/*AARS* versus control, AUC = 0.960 (95% CI; 0.886–1); (**D**) PMP22d versus control, AUC 974 (95% CI; 0.912–1). (**E**) GDF15 in control, mild (CMTES < 10) and severe patients (CMTES ≥ 10). (**F**) GDF15 versus CMTES in CMT patients, with regression shown for all patients and a CMTES < 15 subset. Statistical significant indicated by Student’s *t*-test, where *P < 0.05, **P < 0.01, ***P < 0.001, ****P < 0.0001.

GDF15 levels follow a log-normal distribution for both controls and patients, with a higher degree of variation within the patient population (Fig. 7A). A 2.7-fold increase in GDF15 was seen in CMT patient sera compared to controls (*P* < 0.0001), and similar elevations were observed in the *GARS*/*AARS* (2.5-fold, *P* = 0.005), *PMP22*d (2.1-fold, *P* < 0.0001) groups (Fig. 7A), with a threshold of > 411.8 pg/ml distinguishing CMT patients from controls with a sensitivity of 94% and a specificity of 93%. The two larger genotypic groups, *AARS*/*GARS* and *PMP22*d both had rather tighter distributions than the overall patients, suggesting the genotype has some association with GDF15 level. ROC curves produced to distinguish control versus patient by GDF15 level show a very high level of sensitivity and specificity, with an overall area under curve (AUC) of 0.972 (95% CI; 0.936–1) for all CMT patients (Fig. 7B), with an AUC of 0.960 (95% CI; 0.886–1) for *GARS*/*AARS*, and 0.974 (95% CI; 0.912–1) for PMP22d (Supplementary Fig. 6).

GDF15 was significantly increased in severely affected (CMTES ≥ 10) compared with mildly affected patients (CMTES < 10) (Fig. 7E), and while not significantly correlated with CMTES (Fig. 7F) overall, there was a strong correlation between GDF15 and CMTES for mild-to-moderately affected patients (CMTES < 15, Fig. 7F). GDF15 had a significant positive correlation with age in both healthy control and CMT patient sera, but with a higher rate of increase in CMT patients (Supplementary Fig. 7). Age-adjustment of GDF15 levels to the rate of increase in controls still showed no correlation with CMTES scores (Supplementary Fig. 7).

While GDF15 is not a general marker of myopathy, it has been found to be elevated in mitochondrial myopathy caused by mitochondrial translation defects.^45–47^ Serum GDF15 was analysed from disease-control GNE myopathy patients (*n* = 39) and BMD/DMD adult patients (>20 years, *n* = 34). GNE myopathy displays a similar distal muscle wasting phenotype to CMT, while BMD/DMD have a more generalized and severe muscular dystrophy. GNE myopathy showed a 1.35-fold increase compared to controls (*P* < 0.001), but still significantly lower (413 ng/µl versus 812.4 ng/µl, *P* = 0.0001) compared with CMT patient sera (Fig. 7A). BMD/DMD patients showed decreased GDF15 levels compared to controls (Fig. 7A). ROC curves generated for GNE myopathy have an AUC of 0.802 (95% CI; 0.684–0.920) for differentiation with controls, and an AUC of 0.756 (95%; 0.655–0.857) between GNE myopathy and CMT patients (Fig. 7B–D). ROC curves generated for BMD/DMD have an AUC of 0.723 (95% CI; 0.577–0.869) compared to controls, and an AUC of 0.963 (95% CI; 0.928–0.999) compared with CMT patients. Therefore, we find that strong (2-fold) elevation of GDF15 acts as an effective diagnostic biomarker distinguishing CMT patients from both controls and from two other muscle wasting disorders.

### Complement components and NCAM1 are increased in skeletal muscle of CMT patients

**Figure 8:**
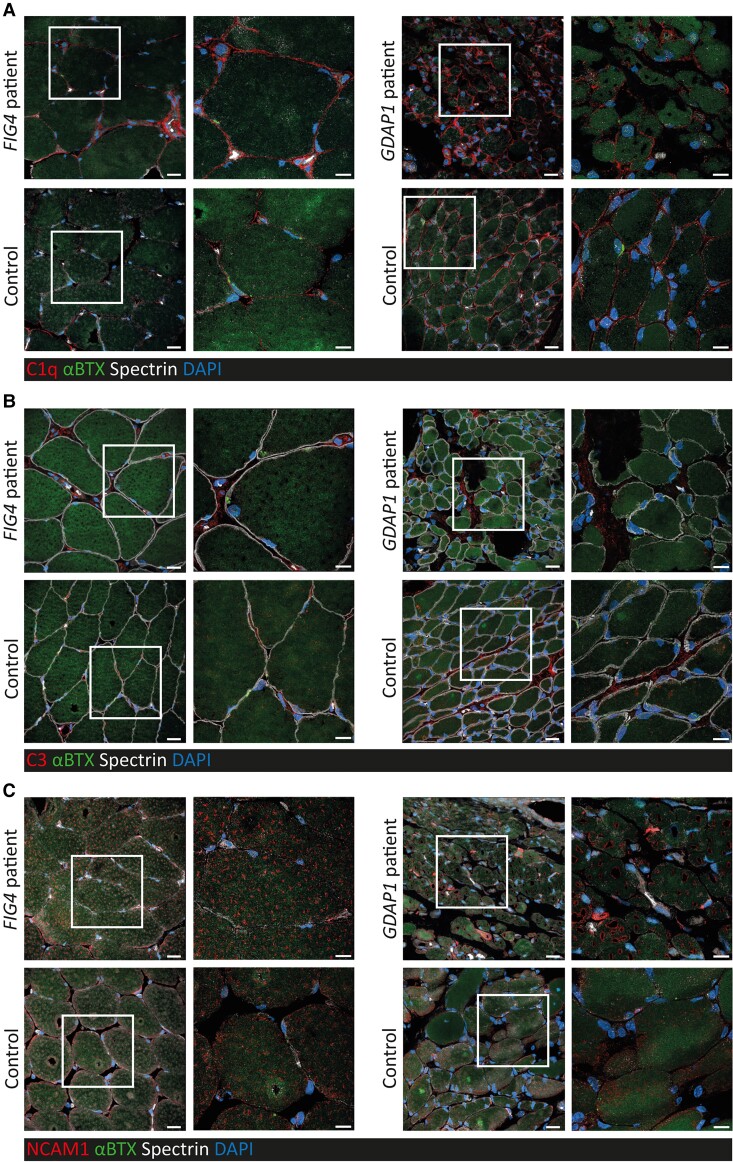
**Complement components and NCAM1 are increased in skeletal muscle of CMT patients.** Confocal microscopy detects more (**A**) complement C1q, (**B**) complement C3, and (**C**) NCAM1-positive structures in muscle biopsies of two patients with CMT due to pathogenic variants in *FIG4* and *GDAP1* compared to age- and sex-matched healthy controls (each in red). In contrast, muscle biopsies from healthy controls show more α-bungarotoxin (BTX, green) positive structures along the basement membrane (Spectrin, grey). Scale bars = 20 µm in overview scans, 10 µm in detail scans (indicated by white box).

To support our findings of increased serum complement components and NCAM1 in CMT we have performed confocal microscopy on skeletal muscle biopsies of two CMT patients carrying pathogenic variants in *FIG4* and *GDAP1*. We detected increased complement C1q, complement C3, and NCAM1 positive structures in muscle biopsies of both patients compared with age and sex matched healthy controls (Fig. 8). These findings are in support of an association between CMT neuromuscular pathology and complement/NCAM1 activation.

### Cell-free mitochondrial-DNA

Several studies have indicated the potential for circulating cell-free mitochondrial DNA (ccf-mtDNA) to be altered in patients with neurodegenerative conditions such as Parkinson’s disease and Friedreich’s ataxia,^48,49^ and similarly to GDF15 and FGF21, ccf-mtDNA has been suggested to be a useful biomarker of mitochondrial disease.^50^ Therefore, we studied ccf-mtDNA in the serum in samples from 42 CMT patients and 42 healthy controls by quantitative PCR. These data (Supplementary Fig. 8) show that there is no difference between control and CMT groups in overall ccf-mtDNA, confirmed by two targeted mtDNA genes (*MT-ND1* and *MT-ND4*). None of the genetic subgroups of CMT show any significant correlation with serum ccf-mtDNA levels.

## Discussion

We applied targeted proteomics to search for biomarkers in a cohort of patients and mouse models with different types of CMT. Our experimental approach enabled us to screen >250 proteins simultaneously in each sample, from which we identified NCAM1 and complement components to be elevated in both patients and mouse models of CMT. We propose that NCAM1 may be a biomarker of CMT with potential capacity to monitor disease-progression, as demonstrated in patients and in several mouse models. Our analysis (ROC curves) shows that NCAM1 can be used as a diagnostic biomarker with good selectivity between CMT patients and controls (Fig. 3A–D). NCAM1 was significantly elevated in all CMT patient analysis subgroups (CMT1, CMT2, *PMP22*d, *GARS*/*AARS*), particularly in *PMP22*d (CMT1A) (Fig. 3A). Ncam1 elevation in mouse models coincided with the development of neuropathic weakness, suggesting that it may be related to the loss of functional axons.^32,51^ For both *Hspb8*^K141N^ and *Gjb1*-null mice, Ncam1 elevation was evident from 6 months, and continued to increase with age in parallel with the progression of the neuropathy (Fig. 2B and C). C61 het mice showed divergent results compared to patients, which may be explained by the variability of the neuropathy and/or other reasons, including sample sizes or other technical issues. It is also possible that Ncam1 elevation is more associated with axonal phenotypes in mouse model, given that our patients have a relatively advanced neuropathy (CMTES; 14.5 ± 3.3, mean ± SD), while in the C61 het mice the neuropathy starts at 2 months and progresses until 10 months of age. In line with the progressive elevation observed in mouse models, NCAM1 was higher in severely affected CMT patients, and showed a significant direct correlation with CMTES (Fig. 3E and F). Furthermore, we have previously shown that both serum Ncam1 levels and clinical symptoms of the neuropathy are restored to wild-type levels following targeted gene therapy in the *Gjb1*-null mice.^31^

Ncam1 may be expressed in Schwann cells,^52,53^ and may cause elevated serum levels in acquired and inherited demyelinating neuropathies including CMT1A,^41^ but may also be expressed in denervated and regenerating muscle fibres^54^ to promote neurite outgrowth and synapse formation.^55^ Muscle NCAM1 expression is high in regenerating muscle and is sustained long after denervation.^56^ Based on the elevation in our axonal mouse models and the lack of increase in pre-symptomatic mice (C61 het), NCAM1 seems more likely to originate from the atrophic or regenerating muscle. Thus, we propose that the observed progressive increase in NCAM1 levels seen in patients and mice is proportional to the degree of muscle denervation and/or regeneration, and thereby is indirectly linked to axon degeneration. In support of this hypothesis we detected increased NCAM1 staining in skeletal muscle of two patients with different genetic forms of CMT (*FIG4*, *GDAP1*) (Fig. 8). We believe that NCAM1 has the potential to reflect clinical severity of neuropathy and may respond to treatments, therefore further investigations are recommended in larger cohorts of patients and in clinical trials.

Our data identified an elevation of the complement system in both axonal and demyelinating CMT. C1q-B and C3 are elevated in *Hspb8*^K141N^ and *Gjb1*-null mice from very early in the pre-symptomatic or mildly symptomatic stage, while levels in *Gars*^C201R^, *Gars*^P278KY^ and C61 het mice were comparable to controls. In CMT patients, C1q-B and C3 were elevated in both axonal and demyelinating forms (Fig. 5A and B). The increase of complement protein levels did not correlate with progression of the phenotype, and similarly in patients there was no relationship with neuropathy severity (Fig. 5C and D and Supplementary Fig. 4) or age (Supplementary Fig. 5).

Deposition of complement proteins, particularly C1q and C3, has been identified at the motor end plates of the neuromuscular junction (NMJ) in both sporadic ALS patients and SOD1-ALS mice, and has been associated with the denervation of muscle endplates.^57,58^ We detected increased C1q and C3 deposition on the sarcolemma, in part co-localized with the NMJs in skeletal muscle biopsies of two patients with different genetic forms of CMT (*FIG4*, *GDAP1*). Additionally, recent data indicate that the NMJ is involved in the pathology of CMT,^59–61^ and C1q-B deposition has been observed in the sciatic nerves of *Sh3tc2*^mut^ mice.^59^ It is possible that complement proteins at the NMJ are involved in analogous processes to C1q- and C3-mediated microglial synaptic elimination processes which occur in the CNS, where complement upregulation is strongly associated with neuron loss.^62^ Therefore, we can suggest that the upregulation of complement proteins in CMT is caused by NMJ degeneration and that serum levels may therefore be reflective of NMJ degeneration. Lack of elevation of serum complement in the *Gars*^mut^ mice is puzzling, given that CMT2D patients did have elevated complement proteins (Fig. 5A and B),^63^ as it has been reported that these mice develop NMJ defects early in the disease course.^63^ It is possible that such processes are already concluded at 5 months of age in these animals.

The role of the complement cascade has been demonstrated in patients and animal models of myasthenia gravis, an autoimmune disease affecting neuromuscular transmission.^64^ Preclinical and clinical studies have confirmed the efficacy of the complement inhibitor Eculizumab, an antibody directed towards C5, which has been recently approved for the treatment of AChR antibody-positive generalised myathenia gravis.^65^ Therapeutic inhibition of the complement system has been shown to be useful also in other neurological diseases. In Alzheimer’s disease, antibody-mediated suppression of C1q has been shown to ameliorate synapse removal in Tau^P301S^ transgenic mice,^66^ while C5 inhibitors are currently being trialled for ALS (ID: NCT0424845). Blockade of terminal complement components have shown success in the treatment of Guillain-Barré syndrome (GBS), an autoimmune condition affecting the peripheral nerves.^67^ Understanding the role of the complement system in the pathomechanism of CMT warrants future work, which will reveal whether complement inhibitor treatments may be of benefit. However, these drugs are highly expensive, therefore the identification of biomarkers predictive of therapeutic response are useful to tailor and monitor complement-directed therapies.

GDF15 is a circulating cytokine and an established biomarker of mitochondrial disorders, sensitive to disease progression and treatment response in these conditions,^46,47,68^ as well as in cardiovascular,^69,70^ kidney^71^ and liver disease,^72,73^ and diabetes mellitus.^74^ We found that serum GDF15 was significantly elevated in all analysed CMT patient subgroups (Fig. 6E), and show that serum GDF15 is highly selective as a diagnostic marker of CMT with a threshold above 417.4 ng/µl giving a sensitivity of 88.9% and a specificity of 92.9% to differentiate symptomatic CMT patients from controls. We observed an early elevation of GDF15 in CMT and a significant relationship to disease severity among mildly affected (CMTES < 15) patients (Fig. 7E and F).

Several studies link GDF15 with peripheral nerve degeneration. Genetic knockout of Gdf15 in mice causes a facial nerve-predominant motor neuropathy and mild metabolic disorder.^75,76^ GDF15 acts as a nerve growth factor, with isolated murine *Gdf15*-null embryonic motor neurons showing reduced viability, which was fully rescued by supplementation of the cell culture media with human GDF15, BDNF or CNTF.^75^ Lesion of the sciatic nerve induces Gdf15 expression in mice,^75^ for which secretion by Schwann cells is the major source, while treatment of the sciatic nerve lesion site with GDF15 promotes regeneration of motor axons and improves lower-limb sensory function,^77^ thus emphasizing the specific function of GDF15 in neurons. Increased serum GDF15 has been reported also in other diseases affecting the peripheral nerves such as hereditary TTR amyloidosis,^78^ and *Gdf15* gene expression is elevated in *Gars*^mut^ CMT mouse models, where its induction was associated with activation of the integrated stress response.^44^ We therefore hypothesise that in CMT GDF15 is secreted to promote regeneration of peripheral neurons following nerve insult, which contributes to elevation of circulatory GDF15 in the serum.

GDF15 levels showed a progressive increase between control, mildly affected and severely affected CMT patients, and a significant direct correlation for mild-to-moderate patients with a CMTES score under 15 (Fig. 7E and F). Unlike NCAM1, GDF15 was greatly increased even in mildly affected CMT patients and increased prior to neuropathy onset in the axonal *Hsbp8*^K141N^ mouse model. This may suggest that GDF15 may be of prognostic value, particularly in axonal CMT; however, longitudinal studies would be necessary to determine this. Whether serum GDF15 levels would be responsive to therapeutic intervention would require further clarification, but given that GDF15 can be elevated at preclinical disease stages it may be that only therapies which fully reverse nerve pathology would cause a response in GDF15.

GDF15 was significantly elevated in CMT in comparison to the non-neuropathy disease controls GNE myopathy and BMD/DMD. While GDF15 levels can still be used to effectively distinguish CMT from GNE myopathy (Fig. 7D), there was a significant elevation of GDF15 within the GNE myopathy cohort compared to controls (Fig. 7A). This is not unexpected, given that GDF15 is increased in other myopathies including sporadic inclusion body myositis (sIBM),^79^ a condition with related pathological and clinical features to GNE myopathy. Serum GDF15, as well as NCAM1, elevation in GNE myopathy likely results from expression by regenerating muscle fibres during the process of progressive degeneration of the overall tissue. We also note that motor axonal neuropathy has been described in patients carrying pathogenic *GNE* mutations, suggesting that nerve involvement in this condition may be an underdiagnosed pathology and may influence clinical presentation or disease progression.^80^ While it is unclear why muscular dystrophy in BMD/DMD would have a different effect on GDF15 levels, decreased GDF15 observed in BMD/DMD patients (Fig. 7A) may be related to administration of steroids, shown to supress serum GDF15.^81^ GDF15 has attracted interest as a biomarker for conditions as variable as mitochondrial myopathy,^46,47,68^ cardiovascular disease,^82^ natural ageing,^70^ type 2 diabetes mellitus,^83^ cognitive decline,^84^ and all-cause mortality.^85^ With no single clear mechanism linking these conditions, it would seem that serum GDF15 expression may be influenced by many separate processes. While GDF15 levels are influenced by age, the degree of GDF15 induction in CMT is so strong that even the youngest patients have higher levels than the oldest controls (Supplementary Fig. 7), making age-adjustment beneficial but unnecessary in CMT. Quantification of circulating cell-free mtDNA, demonstrated that, unlike Parkinson’s disease^48^ and Friedreich’s ataxia,^49^ there is no alteration in CMT patients (Supplementary Fig. 8).

Serum NCAM1 and GDF15 perform well compared to other proposed biomarkers, which is particularly encouraging considering the heterogenous nature of our cohort. Our data indicate that serum GDF15 is a highly selective diagnostic biomarker of CMT (AUC of 0.972, 95% CI; 0.936–1), while NCAM1 (AUC 0.748, 95% CI; 0.6268–0.869) is a more suitable serum biomarker of neuropathy progression. Diagnostic capacity of GDF15 for CMT1A (AUC 0.973, Supplementary Fig. 6) is higher than for plasma TMPRSS5 (AUC 0.913)^20^ and miRNAs *miR-206* (AUC 0.848), *miR-133a* (AUC 0.773), *miR-223-3p* (AUC 0.738).^86^ NEFL is increased in a broad range of neurodegenerative diseases and less specific for CMT (AUC = 0.755). Axonal markers PFN2 and GAMT predict CMT2 also with lower accuracy with ROC AUCs of 0.708 (95%; 0.569–0.846) and 0.693 (95% CI; 0.546–0.839) respectively.^23^ The regression of NCAM1 with CMTES score (*r* = 0.33) was slightly lower than observed for NEFL (*r* = 0.43),^17^ but higher compared to the cutaneous multi-mRNA prediction markers proposed by Fledrich *et al*.^22^ to distinguish mild from severe patients (NCAM1 AUC 0.884 versus 0.74, Supplementary Fig. 2). Combination of several of these markers may offer improved monitoring of disease progression. It should be stressed that such metrics are specific to the patient cohort of the respective study, and replication of each in a fully independent cohort should be considered for comparative validation between these biomarkers.

## Conclusion

In summary, we have identified that NCAM1 and GDF15 are potential biomarkers in a range of CMT subtypes in patients and mouse models. Furthermore, increased levels of components of the complement pathway in CMT patients and mouse models indicates the activation of this inflammatory pathway in early stages of CMT, potentially due to degeneration at the neuromuscular junction. The use of these biomarkers should be considered for preclinical treatment studies in mice, where their response may be indicative of efficacy. Further studies in larger CMT cohorts, including longitudinal studies, should be conducted to validate the clinical value of these biomarkers for clinical and trial use.

## Supplementary Material

awac055_Supplementary_Data
