# Supplementary material for: NCAM1 and GDF15 are biomarkers of Charcot-Marie-Tooth disease in patients and mice
Source: Brain. 2022 Feb 11;145(11):3999–4015. doi: 10.1093/brain/awac055 (PMC9679171; doi:10.1093/brain/awac055)

Supplementary Table 1. LC-MRM protein peptides standards, fold change and p-values of protein peptides in mouse mutant versus wildtype initial screening. See separate Excel file.

Supplementary Table 2. LC-MRM protein peptides standards, fold change and p-values of protein peptides in patient versus control initial screening.

Supplementary Figure 1. ELISA quantification of NCAM1 in mouse model sera.

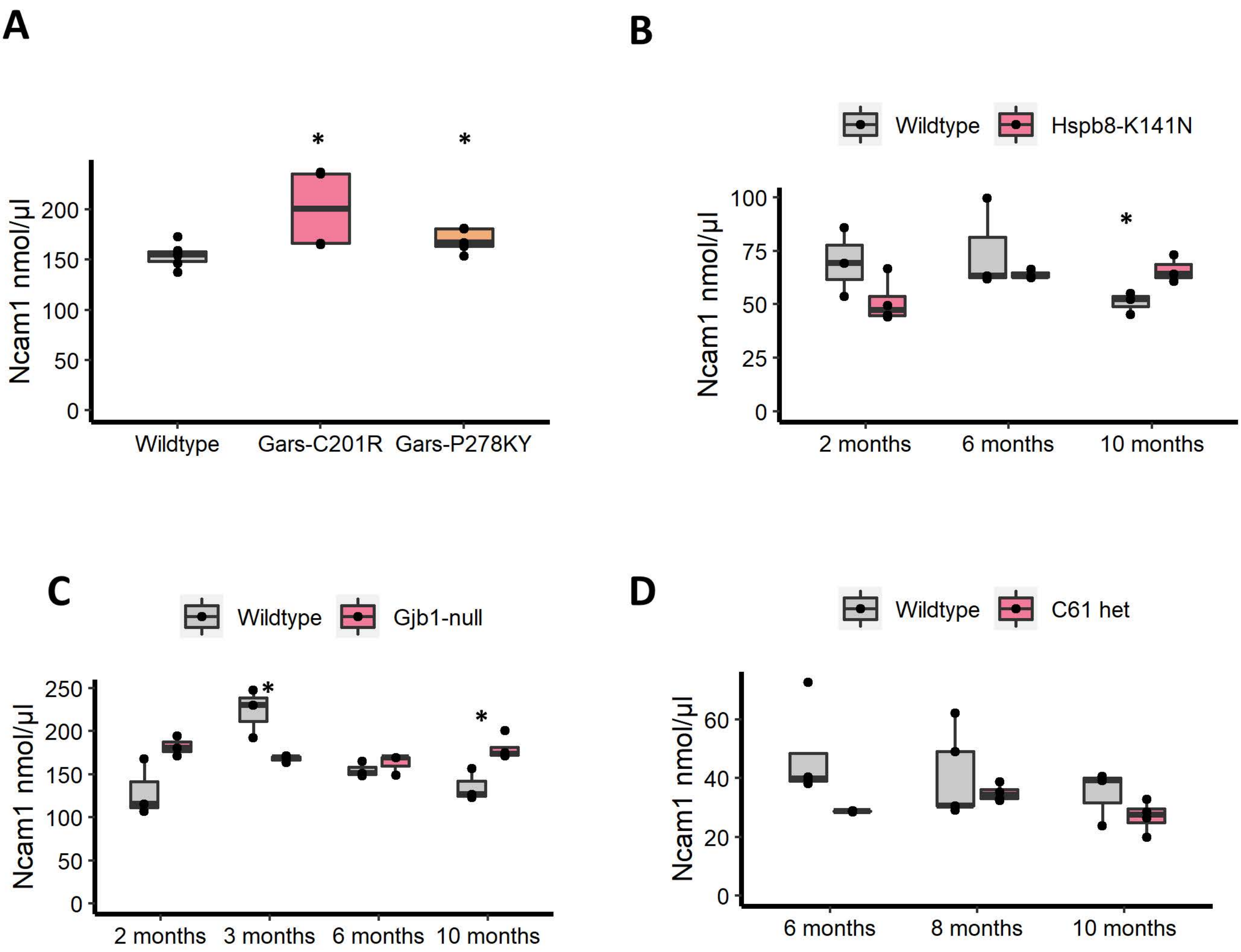

Supplementary Figure 2. NCAM1 versus age in CMT patients and healthy controls. Mild (CMTES < 9) vs severe (CMTES >= 16) ROC curve

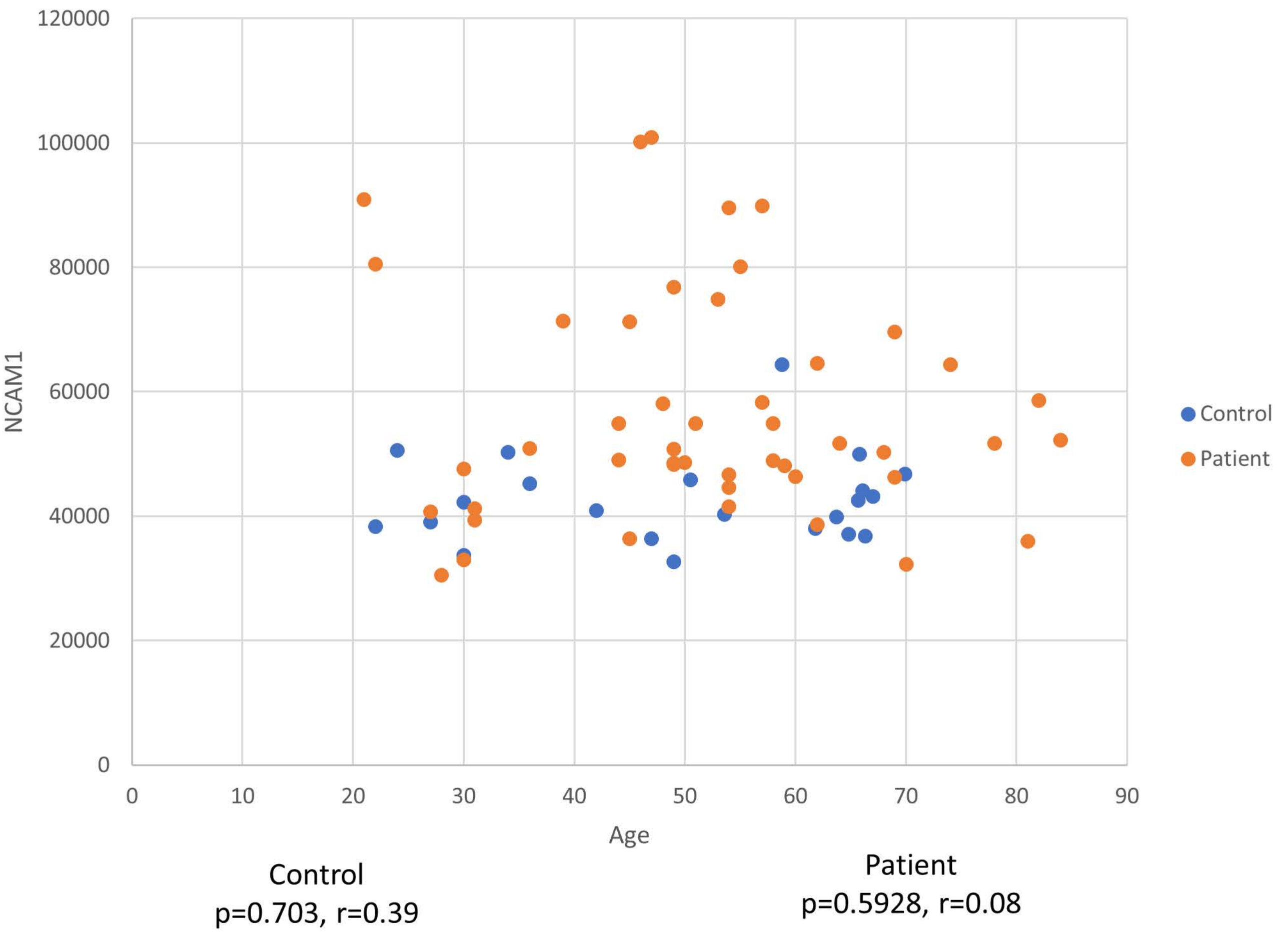

Control
p=0.703, r=0.39

Patient
p=0.5928, r=0.08

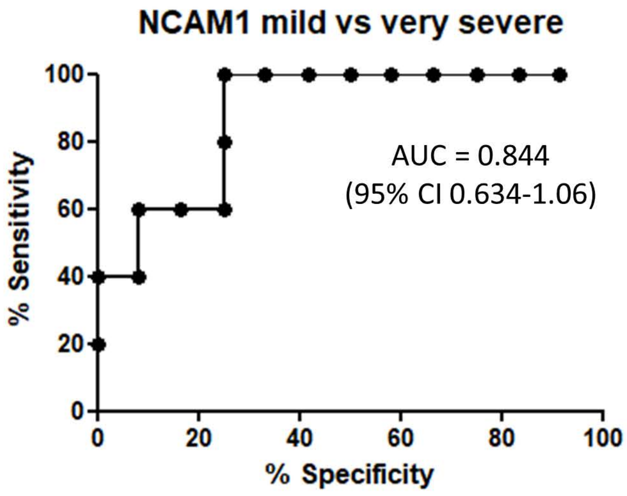

Supplementary Figure 3. Extended Complement protein peptide abundances in mouse models

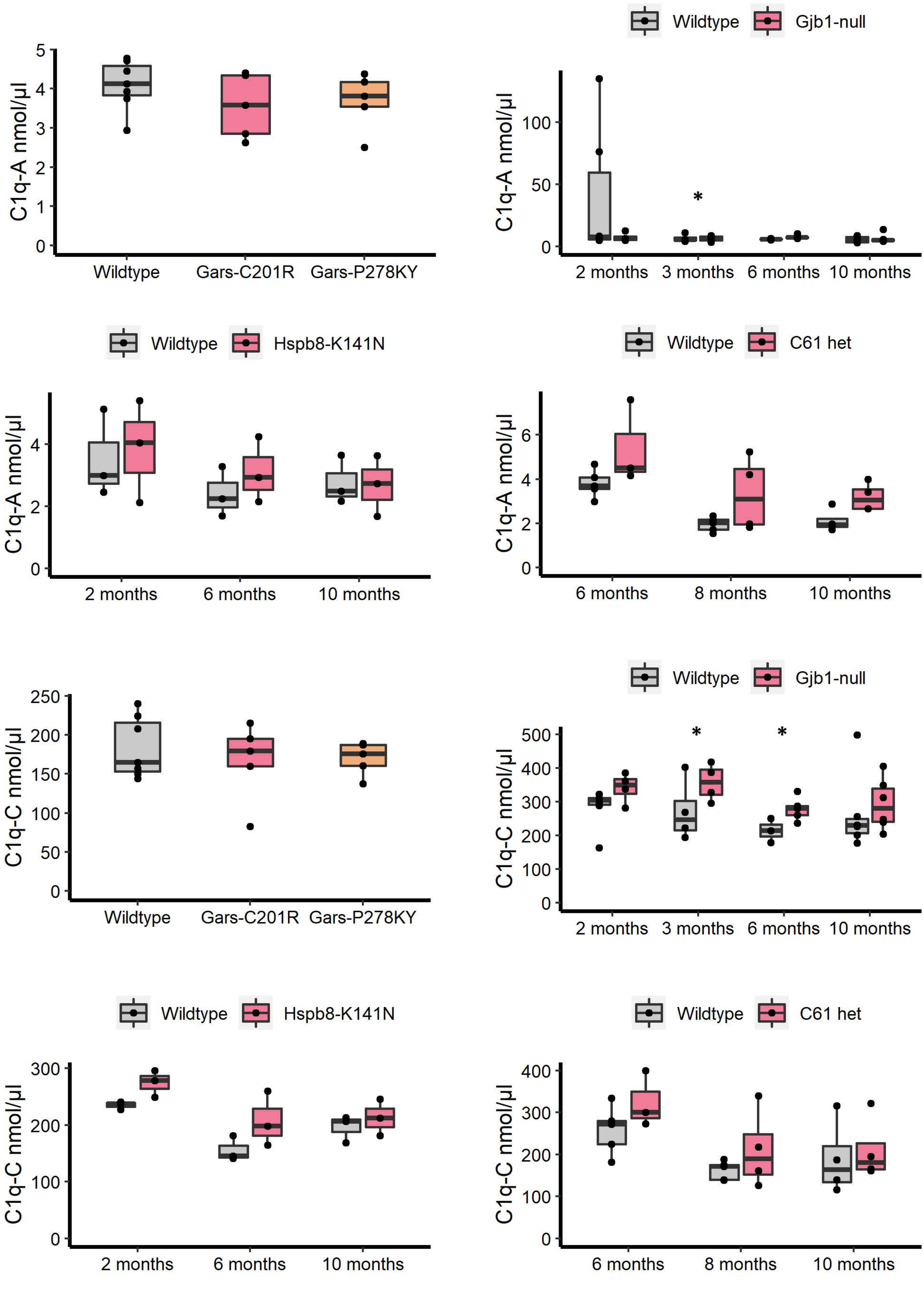

Supplementary Figure 4. Extended Complement protein correlation with CMTES

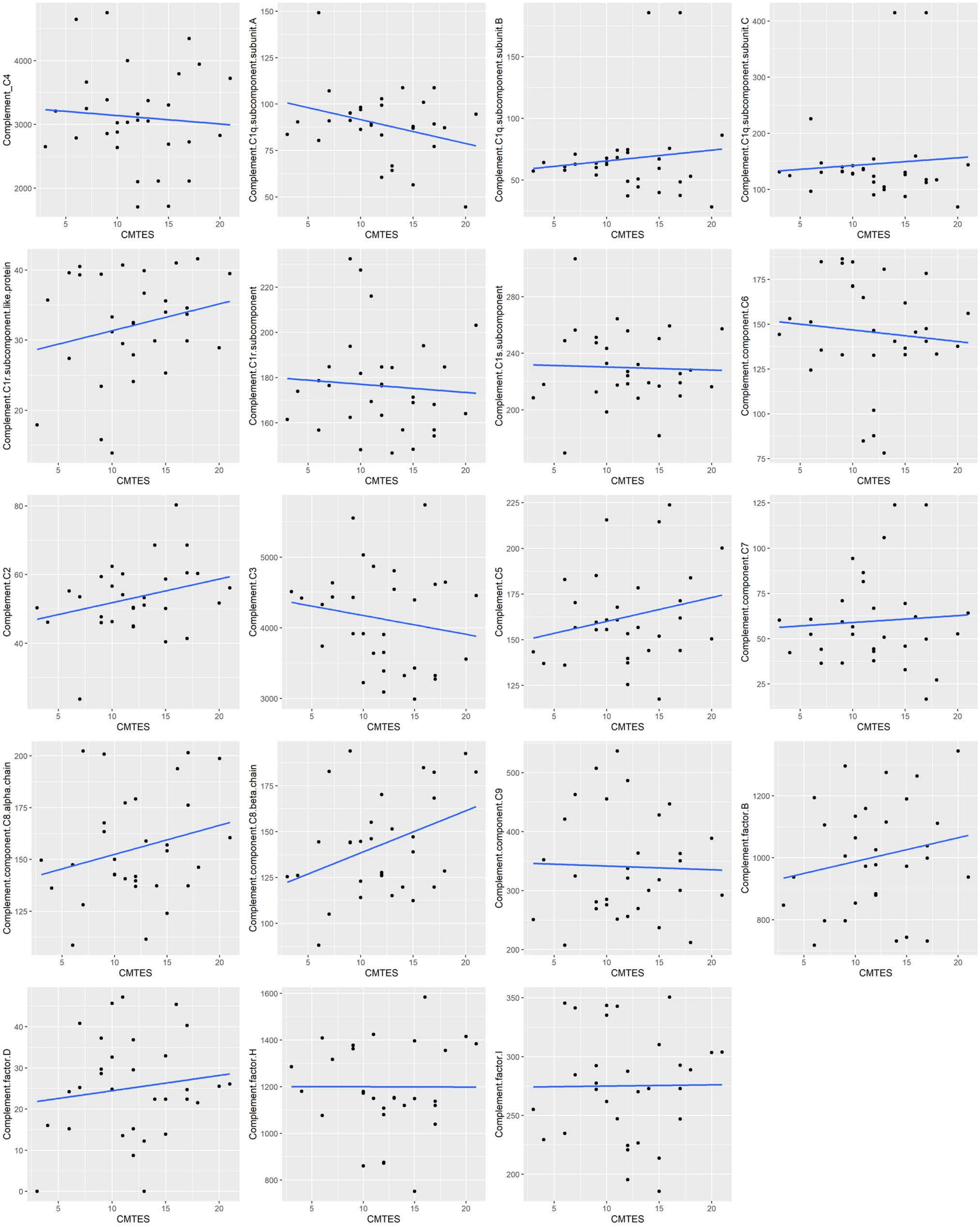

Supplementary Figure 5. C1q and C3 versus age in CMT patients and healthy controls.

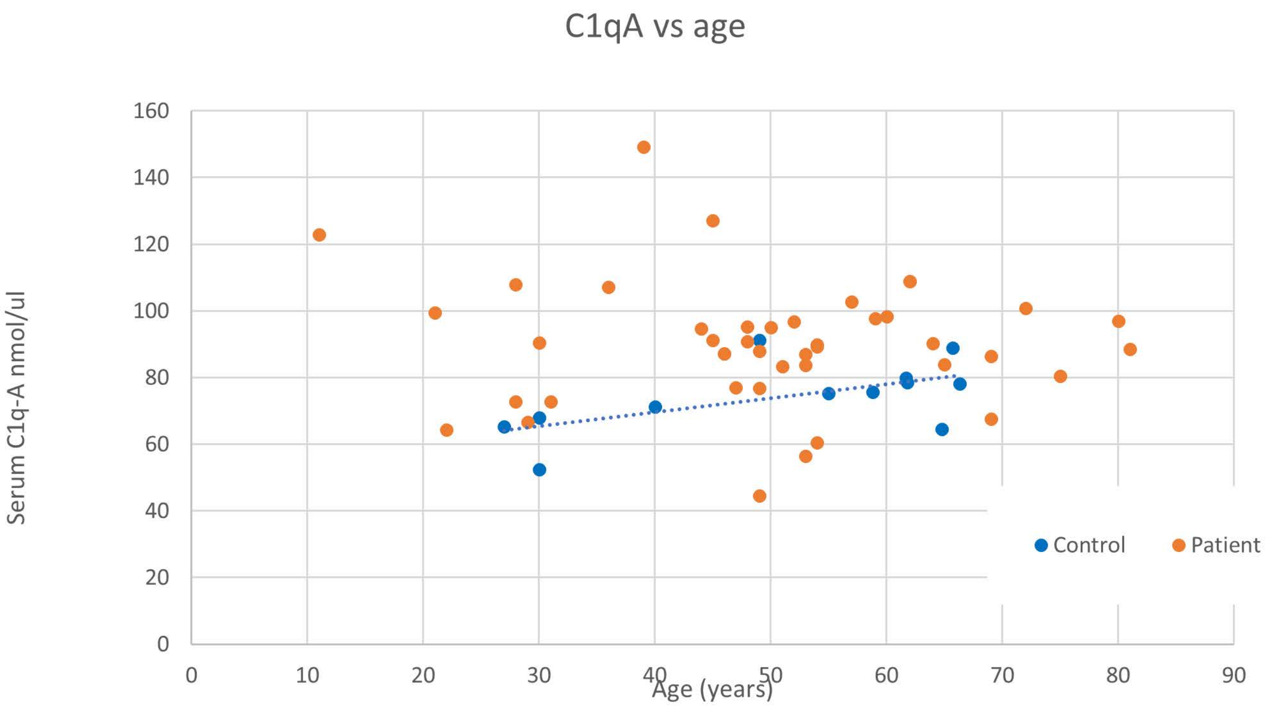

Control
p=0.0409 , r=0. 60

Patient
p=0.7502 , r=0.05

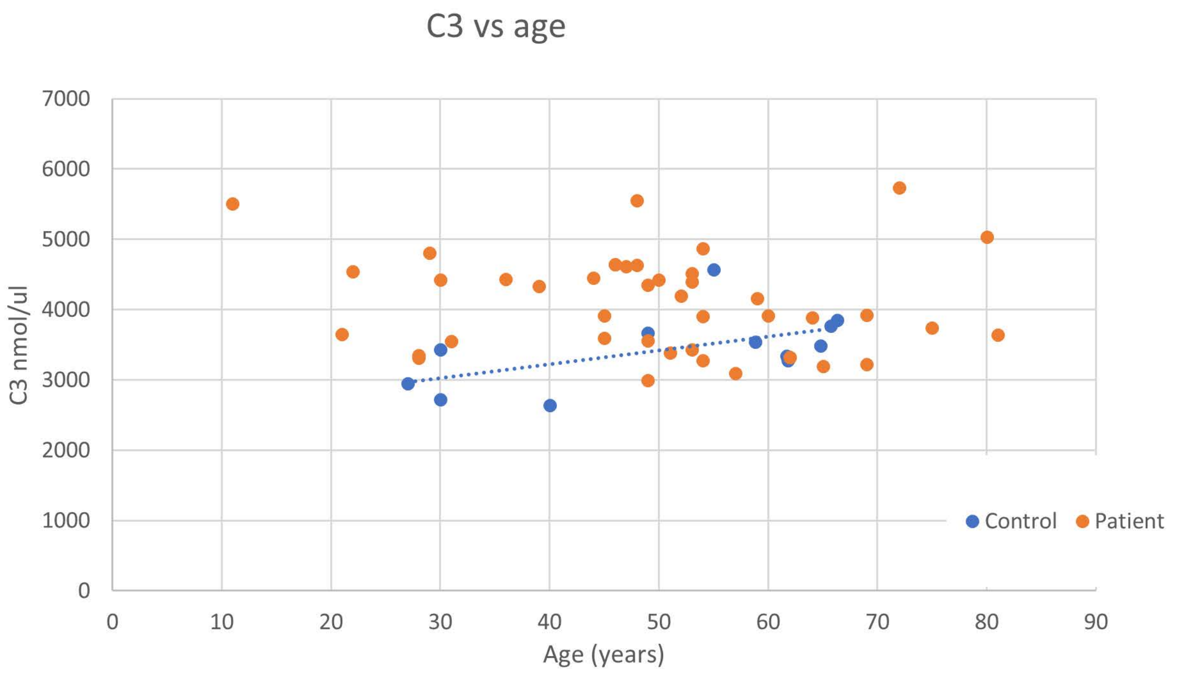

Control
p=0.0550 , r=0. 57

Patient
p=0.3293 , r=0.15

Supplementary Figure 6. ROC curves of GARS/AARS and PMP22d versus control by serum GDF15.

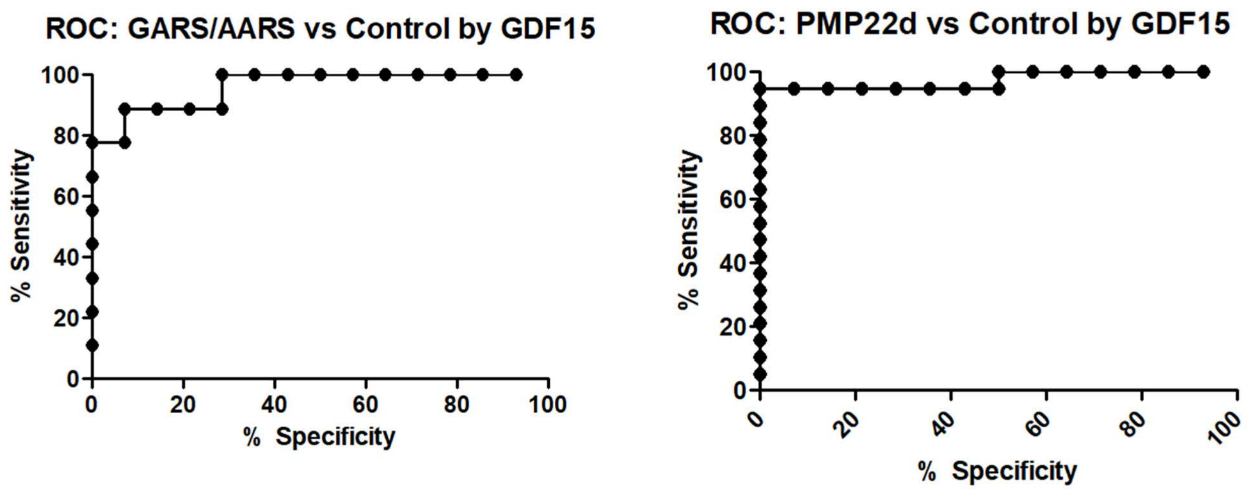

Supplementary Figure 7. GDF15 versus age in CMT patients and healthy controls, and age-adjusted GDF15 versus CMTES

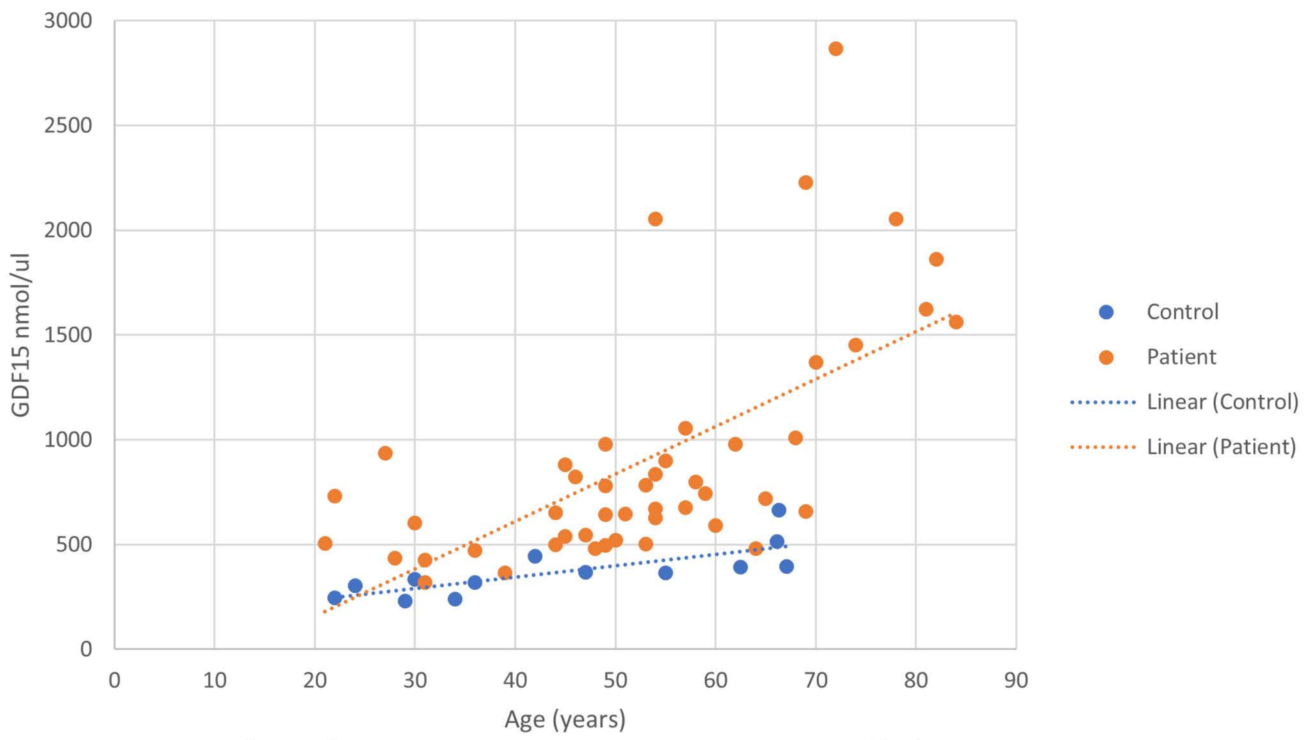

Control
p=0.0021 , r=0.77

Patient
P<0.0001 , r=0.64

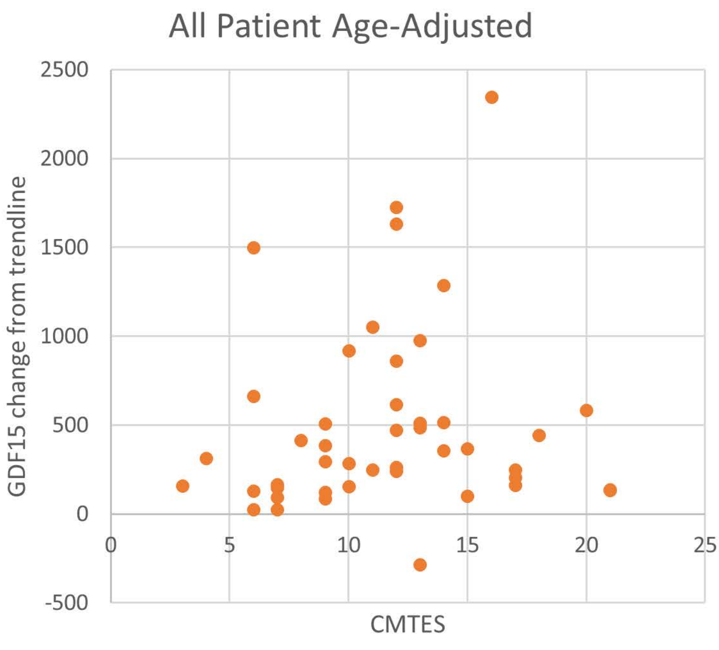

p=0.5110, r=0.10

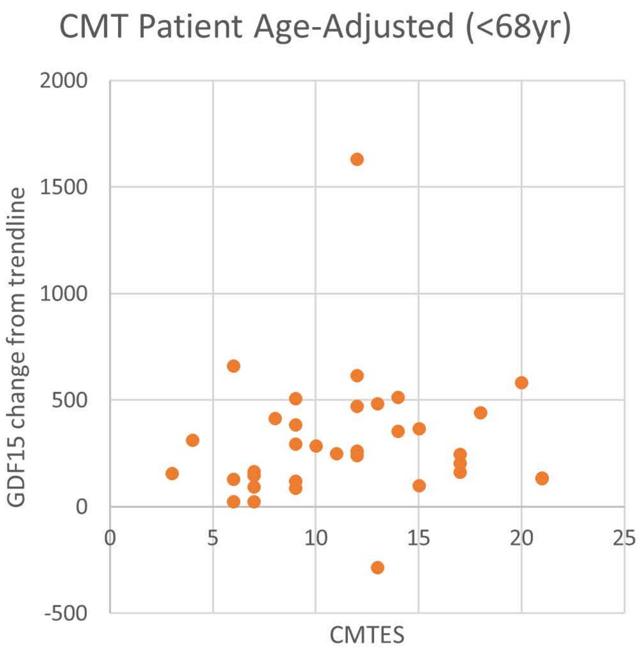

p=0.6384, r=0.08

Supplementary Figure 8. Cell-free mitochondrial DNA in CMT patient serum.

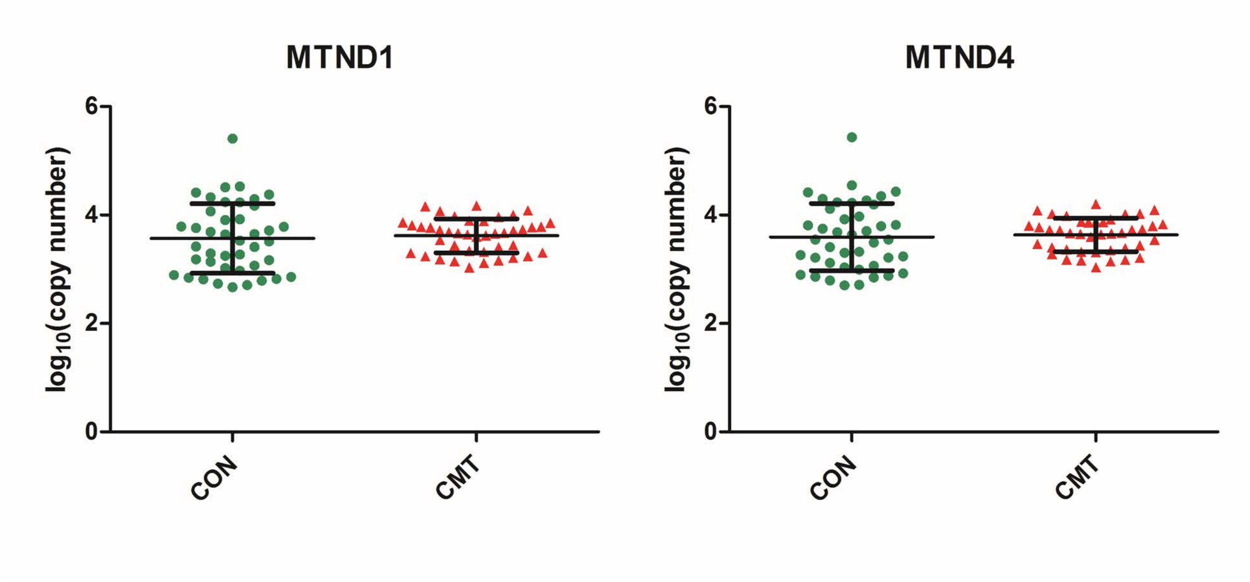

Supplement: awac055_Supplementary_Data [file awac055_supplementary_data.zip › brain-2021-01486-File010.pdf]
