# Supplementary material for: NCAM1 and GDF15 are biomarkers of Charcot-Marie-Tooth disease in patients and mice
Source: Brain. 2022 Feb 11;145(11):3999–4015. doi: 10.1093/brain/awac055 (PMC9679171; doi:10.1093/brain/awac055)
Supplement: awac055_Supplementary_Data [file awac055_supplementary_data.zip › brain-2021-01486-File011.pdf]

Mass spectrometry of human and mouse serum

To control for matrix-induced suppression and other variability in LC-MS, $^{13}C/^{15}N$-labelled peptide analogues are used as internal standards. Peptides were synthesised by Fmoc chemistry, purified by RP-HPLC (reverse phase high performance liquid chromatography) before assessment by MALDI-TOF-MS (matrix assisted laser desorption ionization-time of flight mass spectrometry), characterised by AAA (amino acid analysis) and CZE (capillary zone electrophoresis).

Serum proteolytic digests were prepared on a Tecan Freedom EVO 150 with automated liquid handling and plasma protein preparation (including denaturation, disulphide bond reduction and cysteine alkylation), trypsinisation, SIS peptide addition and SPE (solid phase extraction). For sample preparation 10 µl of serum was subjected to 9 M urea, 20 mM dithiothreitol and 0.5 M iodoacetamide (all in Tris buffer, pH 8.0). Denaturation and reduction was achieved by 30 min incubation at 37°C, with subsequent alkylation by incubation at room temperature for 30 min, avoiding direct light. Proteolysis was performed by addition of TPCK (N-tosyl-L-phenylalanine chloromethyl ketone)-treated trypsin (35 µl at 1 mg/ml; Worthington) at a 20:1 substrate:enzyme ratio, incubated overnight at 37°C before quenching with 1% folic acid (FA). SIS-peptide mixture was then spiked into the digested samples, standard curve samples and quality control (QC) samples and concentrated by solid phase extraction (Oasis HLB, 2 mg sorbent; Waters). Following SPE, the concentrated eluate was frozen, lyophilised and rehydrate in 0.1% FA (final concentration of 0.5 µg/µl digest) for LC/MRM-MS.

Standard curves were prepared by spiking light peptide mixtures into serum tryptic digests. Then peptides were demethylated in order to shift their mass. Nine concentrations are made by serial dilution, such that the highest (upper limit of quantitation, ULOQ) is 1000X the concentration of the lowest (lower limit of quantitation, LLOQ). QC samples were prepared from the same light peptide mix and diluted in dimethylated serum at 4X, 50X and 500X the LLOQ for each peptide.

20 µl injections of the serum tryptic digests were separated with a Zorbax Eclipse Plus RP-UHPLC (reverse phase ultra high performance liquid chromatography) (2.1 x 150 mm, 1.8 µm particle diameter; Agilent) contained within a 1290 Infinity. Peptide separations were achieved at 0.4 ml/min over a 60 min run, by a multi-step LC gradient (2-80% mobile phase B; mobile phase compositions: A was 0.1% FA in H_2O while B was 0.1% FA in acetonitrile). The column was maintained at 40°C. A post-gradient equilibration of 4 min followed each sample analysis.

The LC system was interfaced to a triple quadrupole mass spectrometer (Agilent 6495) via a standard-flow ESI (electrospray ionisation) source, operated in the positive ion mode. General MRM acquisition parameters were: 3.5 kV capillary voltage, 300 V nozzle voltage, 11 l/min drying gas flow at 150°C,

30 psi nebuliser gas pressure, 380 V fragmentor voltage, 5 V cell accelerator potential, and a unit resolution in the first and third quadrupole mass analysers. The high energy dynode multiplier was set to -20 kV, for improved ion detection efficiency and signal-to-noise ratios. Specific LC-MS acquisition on parameters were employed for optimal peptide ionisation/fragmentation and scheduled MRM. Peptide optimisations were optimised previously by direct infusion of the purified SIS peptides. In the quantitative analysis, targets (1 transition per peptide) were monitored over 500 ms cycles with 1 min detection windows.

MRM data was visualised and examined with Skyline Quantitative Analysis software (version 4.1.1.11756, University of Washington). This involved peak inspection to ensure accurate selection, integration and uniformity (of peak shape and retention time) of the SIS and natural peptide forms. After confirming QC criteria (1/x regression weighting, <20% deviation in the QC's level's accuracy) the standard curve was used to calculate the peptide concentration in fmol/ul of serum in serum samples by linear regression.

Fluorescent labelling of cryo-embedded muscle sections

10µm thick sections of cryo-embedded muscle biopsies were fixed with 4% PFA/PBS (pH =7.4) for 15 minutes at room temperature (RT). Fixed sections were blocked with 1% BSA/1%DMSO/PBS for 1h at RT. Three individual staining panels for NCAM1, Complement C1q and Complement C3 were performed. For NCAM1-staining a primary antibody cocktail containing anti-NCAM1 (1:100, Abcam, Cat# ab9018), anti-α-II-Spectrin (1:100, Invitrogen, Cat# PA5-44905), and α-Bungarotoxin-AlexaFluor647 (1:100, Invitrogen, Cat# B35450) in blocking buffer was used. The second antibody cocktail consisted of anti-complement C1q (1:50, Life Technologies, Cat# MA183963), anti-α-II-Spectrin (1:100, Invitrogen, PA5-44905), and α-Bungarotoxin-AlexaFluor647 (1:100, Invitrogen, Cat# B35450) in blocking buffer. The third antibody cocktail consisted of anti-complement C3 (1:50, Invitrogen, Cat# PA5-21349), anti-Spectrin (1:100, Novocastra, Cat# NCL-SPEC1), and α-Bungarotoxin-AlexaFluor647 (1:100, Invitrogen, B35450) in blocking buffer. The sections were incubated with the respective antibody cocktails for 4h at RT in the dark, followed by washing 3 steps with blocking buffer for 15 min each. Sections stained with the first or second antibody cocktail were stained for 4h at RT with a secondary antibody cocktail of goat anti-mouse AlexaFluor 488 (1:200, Biolegend, Cat# 405319), donkey anti-rabbit AlexaFluor-plus555 (1:100, Invitrogen, Cat# A32794) and DAPI (1:500, Carl Roth, Cat# 6335.1) in blocking buffer. Sections stained with the third antibody cocktail were stained for 4h at RT with donkey anti-rabbit AlexaFluor-plus488 (Invitrogen, Cat# A32790, 1:100), goat anti-mouse AlexaFluor488 (Biolegend, Cat# 405324), and DAPI (1:500, Carl Roth, Cat# 6335.1) in blocking buffer. All sections were washed 3 times with blocking buffer for 15min each and stained with DAPI (Carl Roth, Cat# 6335.1, 1mg/ml, 1:500) in blocking buffer for 10min at

RT. Finally, all sections were washed 2 times with blocking buffer, and one time with water for 15min each at RT and covered with fluorescence mounting medium (Agilent Technologies, Cat# S302380-2).

Confocal laser scanning microscopy (CLSM) and image processing of fluorescent-labelled muscle sections

For high-resolution microscopy of fluorescent-labelled muscle sections a Leica TCS SP8 confocal laser scanning microscope with acousto-optic tuneable filters, an acousto-optical beam splitter, internal hybrid detectors (HyD SP), and a LMT200 high precision scanning stage was used. Imaging of coverslip-embedded samples was performed via a Leica HC PL APO 63x/1.20 W CORR objective combined with digital zoom factors 0.75 or 1.75. Fluorescence signals were generated via sequential scans, exciting AlexaFluor488 labelled structures via an argon laser at 488nm and detecting with an internal HyD at 500–550nm. AlexaFluor555 labelled structures were excited by a diode-pumped solid-state laser at 561nm and detected with internal HyDs at 600–650nm. The third sequence for visualizing AlexaFluor647 labelled structures involved a 633nm helium-neon laser for excitation and internal HyD at 650-700nm for detection. In the last sequential scan DAPI was excited via a 405nm diode-pumped solid-state laser and detected by an internal HyD at 450-500nm. Generated images were deconvoluted with Huygens Professional (SVI) and reconstructed with Imaris software (Bitplane).
